# Contractile and Genetic Characterization of Cardiac Constructs Engineered from Human Induced Pluripotent Stem Cells: Modeling of Tuberous Sclerosis Complex and the Effects of Rapamycin

**DOI:** 10.3390/bioengineering11030234

**Published:** 2024-02-28

**Authors:** Veniamin Y. Sidorov, Tatiana N. Sidorova, Philip C. Samson, Ronald S. Reiserer, Clayton M. Britt, M. Diana Neely, Kevin C. Ess, John P. Wikswo

**Affiliations:** 1 Vanderbilt Institute for Integrative Biosystems Research and Education, Vanderbilt University, Nashville, TN 37235, USAclayton.britt@vanderbilt.edu (C.M.B.); john.p.wikswo@vanderbilt.edu (J.P.W.); 2 Department of Biomedical Engineering, Vanderbilt University, Nashville, TN 37235, USA; 3 Department of Anesthesiology, Vanderbilt University Medical Center, Nashville, TN 37232, USA; 4 Department of Physics and Astronomy, Vanderbilt University, Nashville, TN 37212, USA; 5 Department of Pediatrics, Vanderbilt University Medical Center, Nashville, TN 37232, USA; diana.neely@vumc.org (M.D.N.); kevin.ess@vumc.org (K.C.E.); 6 Department of Molecular Physiology and Biophysics, Vanderbilt University, Nashville, TN 37232, USA

**Keywords:** tuberous sclerosis complex, hiPSC, mTOR, rapamycin, I-Wire cardiac construct, heart-on-a-chip

## Abstract

The implementation of three-dimensional tissue engineering concurrently with stem cell technology holds great promise for in vitro research in pharmacology and toxicology and modeling cardiac diseases, particularly for rare genetic and pediatric diseases for which animal models, immortal cell lines, and biopsy samples are unavailable. It also allows for a rapid assessment of phenotype–genotype relationships and tissue response to pharmacological manipulation. Mutations in the *TSC1* and *TSC2* genes lead to dysfunctional mTOR signaling and cause tuberous sclerosis complex (TSC), a genetic disorder that affects multiple organ systems, principally the brain, heart, skin, and kidneys. Here we differentiated healthy (CC3) and tuberous sclerosis (TSP8-15) human induced pluripotent stem cells (hiPSCs) into cardiomyocytes to create engineered cardiac tissue constructs (ECTCs). We investigated and compared their mechano-elastic properties and gene expression and assessed the effects of rapamycin, a potent inhibitor of the mechanistic target of rapamycin (mTOR). The TSP8-15 ECTCs had increased chronotropy compared to healthy ECTCs. Rapamycin induced positive inotropic and chronotropic effects (i.e., increased contractility and beating frequency, respectively) in the CC3 ECTCs but did not cause significant changes in the TSP8-15 ECTCs. A differential gene expression analysis revealed 926 up- and 439 down-regulated genes in the TSP8-15 ECTCs compared to their healthy counterparts. The application of rapamycin initiated the differential expression of 101 and 31 genes in the CC3 and TSP8-15 ECTCs, respectively. A gene ontology analysis showed that in the CC3 ECTCs, the positive inotropic and chronotropic effects of rapamycin correlated with positively regulated biological processes, which were primarily related to the metabolism of lipids and fatty and amino acids, and with negatively regulated processes, which were predominantly associated with cell proliferation and muscle and tissue development. In conclusion, this study describes for the first time an in vitro TSC cardiac tissue model, illustrates the response of normal and TSC ECTCs to rapamycin, and provides new insights into the mechanisms of TSC.

## 1. Introduction

There are many challenges in understanding and treating rare diseases, particularly those with pediatric onset and not highly replicative animal models. Microphysiological systems (MPSs), also known as organs-on-chips, allow for the study of three-dimensional tissue constructs generated with human cells that are cultured in environments that are more realistic than biology on plastic [1]. The challenge then becomes the sourcing of the cells. For induced diseases, for example those associated with infection or inflammation, one might start with normal primary cells or immortal cell lines to grow a tissue construct or an organ-on-a-chip, and then observe the effects of disease induction and treatment. With pediatric onset and rare genetic diseases, cadaver or biopsy-derived primary cells or immortalized cell lines are a rarity. With the advent of somatic cell reprogramming techniques for the induction of human pluripotent stem cells (hiPSCs) and techniques for the controlled differentiation of hiPSCs into specific organotypic cells, such as cardiomyocytes, it is now possible to generate, albeit with significant effort and expenses, patient-derived hiPSC lines that can then be differentiated into one or more desired cell types [2,3]. Given the effort and costs currently required for generating and validating an iPSC disease-model-on-a-chip, one must be careful in selecting the disease and the organ to recapitulate using MPS approaches. This paper describes one such evaluation.

Tuberous sclerosis complex (TSC) is an autosomal dominant neurogenetic disorder that has variable clinical manifestations and occurs due to loss-of-function mutations in the *TSC1* or *TSC2* genes [4]. In the cardiovascular system, TSC is characterized by the abnormal growth of cardiac tissue in the ventricular and septal walls, cardiac rhabdomyomas, and arrhythmias, which may or may not be associated with tumors [5,6,7]. Large tumors can also lead to hemodynamic compromise, resulting in congestive heart failure [7]. Since the mechanistic target of rapamycin (mTOR) pathway appears to play an important role in the pathogenesis of TSC, modification of mTORC1 and/or mTORC2 by rapamycin and similar drugs (rapalogs) could have great therapeutic potential. A variety of animal TSC models have been developed [8,9,10]; however, these do not accurately recapitulate human pathology, and clinical trials with rapalogs have not been as successful as predicted from animal experiments [11,12].

Rapamycin was originally thought to be an anti-fungal antibiotic isolated from *Streptomyces hygroscopicus* [13]. Through interactions with the cytosolic immunophilin FKBP12, rapamycin specifically inhibits a serine/threonine protein kinase found in both mTOR complex 1 (mTORC1) and mTOR complex 2 (mTORC2) [14]. These protein complexes have distinct sensitivities to various signaling and metabolic factors. The rapamycin-FKBP12 complex directly inhibits mTORC1, but not mTORC2 [15]. The activity of mTORC1 is involved in many vital cellular processes, including the regulation of growth, proliferation, differentiation, autophagy, and metabolism, in response to changes in environmental conditions and/or stress responses. mTORC2 presumably regulates survival and polarity [16,17,18,19].

Rapamycin has been found to improve cardiac function under different pathological conditions [20,21]. In particular, rapamycin reduced heart weight in a murine model of induced hypertrophy [22]. Treatment with rapamycin decreased hypertrophy in a phenylephrine model in vitro and improved cardiac function in a rat model of aortic banding hypertrophy [23]. In a model of infarction-induced heart failure, rapamycin was demonstrated to prevent CM apoptosis, promote autophagy, and attenuate myocardial fibrosis [24]. In neonatal rat CMs, rapamycin reduced the elevated HIF-1α transcription factor expression at an early stage of hypoxic preconditioning (HPC) and lessened the HPC cardioprotective effect [25]. Recent findings emphasize the potential role of the mTOR pathway in the pathogenesis of atherosclerosis and suggest rapamycin and its highly selective analogs as promising therapeutic approaches [26,27,28].

Three-dimensional engineered cardiac tissue constructs (ECTCs) present a unique opportunity to conduct pharmacological and toxicological studies and model cardiac function and diseases in vitro [29,30]. Patient-derived pluripotent stem cells can provide an unlimited source of cardiomyocytes (CMs) for cardiac tissue engineering. To create ECTCs, differentiated CMs are dissociated and encapsulated within different biomaterials to provide a three-dimensional microenvironment. This procedure disrupts cell-to-cell electrical and mechanical coupling and involves additional steps to handle the cells. In this work we utilized our “I-Wire” heart-on-a-chip platform [31,32,33] to differentiate iPSCs and grow ECTCs in the I-Wire ECTC mold without the need to dissociate the CMs before the formation of the ECTC. To demonstrate this process, we used normal (CC3) and disease-specific (TSP8-15) human induced pluripotent stem cell (hiPSC) lines, characterized their contractile and elastic properties, and investigated the effect of the rapamycin on mechano-elastic properties and gene expression patterns.

As animal experimental models do not reproduce human physiology in the case of TSC and there is a desire to minimize animal experiments, there is a pressing need for in vitro models of disease. While models using human stem cell technology are usually two-dimensional or include the dissociation of differentiated cardiomyocytes as a mandatory intermediate step, we hypothesize that our 3D I-Wire TSC model, which is constructed from directly differentiated CMs from hiPSCs, could recapitulate aspects of human cardiac TSC pathology more closely than other models, reproduce the functional abnormalities in contractility caused by *TSC2* deficiency, and more carefully replicate changes in the mechano-elastic properties and gene expression in response to treatment with rapamycin.

The motivation for the work reported herein was based on the facts that (i) TSC animal models do not replicate human physiology and, as a consequence, are unable to accurately reproduce the human pathology, (ii) clinical trials with rapalogs have not been as successful as predicted from animal experiments, (iii) heart-on-a-chip models allow for studying in vitro the disease mechanisms responsible for the mechanical and electrical activity disturbances in the human heart, and (iv) as compared with models using human cells on plastic, 3D models allow for replication to certain degrees of the complex microenvironment in the heart and integration of multiple cell types in a spatially organized manner.

This work addresses the first step in the development and validation of a cardiac MPS model for TSC and its treatment, demonstrating that an hiPSC MPS cardiac model can distinguish between four distinct situations: cells with wild-type versus mutant genotypes, and drug-treated versus vehicle-treated. These results establish the utility of this model and enable future detailed and laborious investigations to identify specific mechanisms of action of the drug [34,35] on specific cells of the heart [36].

## 2. Materials and Methods

### 2.1. Preparation of PDMS Casting Mold and Cell Mixture

**Figure 1:**
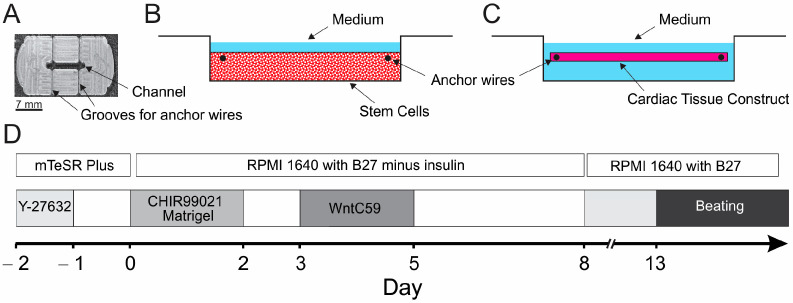
Procedure for creating engineered cardiac tissue constructs (ECTCs). (**A**) Photograph of the mold. (**B**) Mold filled with fibrinogen-based matrix and iPSCs. (**C**) Differentiated cardiomyocytes self-organized into an ECTC suspended between two stationary wires. (**D**) Schematic representation of cardiomyocyte differentiation protocol.

A polydimethylsiloxane (PDMS) mold with 0.25 mm-diameter titanium wires was utilized as a horizontal support for the ECTCs [31]. To prepare the mold, a template with six cavities was made from monolithic acrylic plastic. Two thin edges of the same material were inserted into each cavity to form channels for the supporting titanium wire. The cavities were filled with liquid PDMS mixed with a hardener (10:1) and degassed. The final PDMS matrix had a channel with a depth of 2 mm, a width of 2 mm, and a length of 10 mm, as well as two grooves to accommodate the anchor wire (Figure 1A). Each PDMS mold was transferred to the well of a 6-well plate, glued to the bottom using liquid PDMS, and sterilized by UV irradiation for 30 min. To reduce cell adhesion, the channels in the PDMS devices were treated for 60 min with 0.2% Pluronic^®^ F-127 (MilliporeSigma, Burlington, MA, USA).

### 2.2. Derivation and Validation of hiPSCs

The hiPSC lines used for this study (CC3 and TSP8-15) were derived and validated according to our established protocols [37,38,39]. In brief, dermal fibroblasts were obtained by skin biopsy after appropriate patient consent/assent under the guidelines of an approved IRB protocol (Vanderbilt No. 080369). About 6 × 10^5^ fibroblasts were reprogrammed by electroporation with CXLE plasmid vectors using the Neon Transfection System (Life Technologies, Carlsbad, CA, USA), following published methods [40], and then plated at 5 × 10^4^ cells/well into 6-well plates coated with Growth Factor Reduced (GFR) Matrigel™ (BD Biosciences, Franklin Lakes, NJ, USA). Two days later, the cells were transferred into TeSR-E7 medium (#05919, #05914; STEMCELL Technologies, Vancouver, BC, Canada) and maintained until hiPSC colonies were ready to be manually isolated (about 4 weeks) and propagated in mTeSR medium (#85851, #85852; STEMCELL Technologies, Vancouver, BC, Canada). The lack of plasmid integration into the genomic DNA was demonstrated by qPCR, karyotype analyses were performed using standard protocols with at least 20 metaphase spreads (Genetics Associates, Nashville, TN, USA), and pluripotency was validated by PluriTest [41], immunocytochemistry, and the capacity of the hiPSCs to differentiate into cells of the three germ layers. DNA sequencing confirmed a nonsense heterozygous mutation in exon 31 of the *TSC2* gene.

### 2.3. hiPSC Differentiation

The hiPSCs maintained in mTeSR Plus medium were dissociated into a single cell suspension using an Accutase^TM^ protocol (07920, STEMCELL Technologies, Vancouver, BC, Canada) and centrifuged for 5 min at 200 g. The cell pellet was resuspended in medium (mTeSR Plus, 100-0276, STEMCELL Technologies, Vancouver, BC, Canada), and the cell density was adjusted to 2 × 10^7^ cells/mL. To 500 μL of this cell suspension, the following components were added: 100 μL of fibrinogen (20 mg/mL, stock solution, Sigma-Aldrich, St. Louis, MO, USA), 33 μL of aprotinin (1 mg/mL, stock solution, Abcam, Waltham, MA, USA), 100 μL of GFR Matrigel™ (stock solution, BD Biosciences, Franklin Lakes, NJ, USA), and 10 μM ROCK inhibitor Y-27632 (MedKoo Biosciences, Inc., Morrisville, NC, USA). Then, the cell suspension was diluted with 260 μL of mTeSR Plus medium. At the end, 7 μL of thrombin (100 U/mL, Sigma-Aldrich, St. Louis, MO, USA) was added, and the final cell density was 10^7^ cells/mL. Next, 100 µL of this final cell suspension was pipetted into the channel of each PDMS device (Figure 1B) and incubated at 37 °C and 5% CO2 for one hour. After polymerization of the fibrin, 4 mL of mTeSR Plus medium containing aprotinin (33 μg/mL) and ROCK inhibitor (10 μM) (MedKoo Biosciences, Inc., Morrisville, NC, USA) was added to each well. After encapsulation of the hiPSCs, the Matrigel^TM^/fibrinogen-based matrix condensed over time while the hiPSCs formed growing clusters (Figure S1).

Thereafter, hiPSCs were differentiated into CMs directly in a 3D chamber in the PDMS casting mold (Figure 1B,C). To differentiate CMs, we applied a modified small-molecule protocol [42,43,44,45,46,47,48] (Figure 1D) that is based on the modulation of the Wnt/β-catenin signaling pathway. This pathway is actively involved in the regulation of hiPSCs and plays a crucial role in both maintaining stem cell pluripotency and the differentiation of hiPSCs. Briefly, CHIR-99021 selectively inhibits GSK-3β, increases the cytosolic levels of β-catenin, and thereby activates the Wnt/β-catenin signaling pathway. Activation of the Wnt/β-catenin signaling pathway leads to expression of the genes associated with cardiac development and promotes mesoderm induction, which is a critical step towards differentiating into the cardiomyocyte lineage. Following the application of Wnt-c59, the Wnt/β-catenin pathway is inhibited to induce cardiomyocyte differentiation from the mesoderm [49,50].

Two days after seeding the hiPSCs into the PDMS molds, differentiation was initiated on day 0 by replacing mTeSR Plus medium (STEMCELL Technologies, Vancouver, BC, Canada) with RPMI 1640 (Gibco^TM^, ThermoFisher Scientific, Waltham, MA, USA) supplemented with B-27 without insulin, a serum-free supplement containing 20 different components, including BSA, vitamins, fatty acids, and others (minus insulin, A1895601, ThermoFisher Scientific, Waltham, MA, USA), CHIR-99021 (6 μM, AdooQ BioScience, Irvine, CA, USA), and 2% Matrigel™ (GFR, BD Biosciences, USA) (Figure 1D). On day 2, CHIR99021 and Matrigel™ were removed from the medium. After a resting period of 24 h, the cells were treated with Wnt-C59 (2 μM, Tocris Bio-Techne, Minneapolis, MN, USA) for two days. On day 5, Wnt-C59 was removed. As of day 8, the cultures were maintained in RPMI supplemented with B-27 plus insulin (17504044, ThermoFisher Scientific, Waltham, MA, USA). The first spontaneous ECTC contractions were observed after 13 days of differentiation, and the contractions gradually increased over time. The measurements of contractility and elasticity were conducted on days 28–35 of differentiation.

### 2.4. Data Registration and Processing

The I-Wire system has been described in detail in our previous work [31]. In brief, the I-Wire setup is stationed on an inverted optical microscope (Eclipse Ti, Nikon, Melville, NY, USA) equipped with a digital camera system (Zyla sCMOS Camera, Andor Technology, Belfast, Northern Ireland). The stretching lateral force, which was applied to the ECTC via a flexible probe, was adjusted by means of a precisely controlled motorized stage (MS-2000 Flat-Top XYZ Automated Stage, ASI, Eugene, OR, USA). Movies, 12–15 s in duration, were acquired at 160–200 frames per second (Supplementary Materials). The spring-like characteristic of the flexible probe was calibrated using an analytical balance (Torbal, AGCN100, Scientific Industries Inc., Bohemia, NY, USA) and a high-precision micromanipulator (Newport, Irvine, CA, USA).

Processing and analysis of the optical recordings of contracting ECTCs were conducted using MATLAB (R2023b, MathWorks, Natick, MA, USA). First, the recorded data were binarized, then the coordinates of the centroid were detected in each image and contraction trace was reconstructed. Based on the geometry of the probe placement and probe tip location, the exerted force during systole and diastole, the developed force as a difference between the force corresponding to tip positions during maximal contraction, and the minimal force during relaxation were calculated [31,33]. Diagrams of the registration system and force vectors and a description of the modified strength/stress equation for calculation of elasticity are presented in the Supplementary Materials (Figures S2 and S3).

### 2.5. RNA Preparation, Next-Generation Sequencing (NGS), and Gene Set Enrichment Analysis (GSEA)

CC3 and TSP8-15 ECTCs were treated with 10 nM rapamycin (R8781, Sigma-Aldrich, St. Louis, MO, USA) for two days, the ECTCs were dissociated, and total RNA was extracted using the RNeasy Mini Kit (QIAGEN, Germantown, MD, USA) in accordance with the manufacturer’s instructions. Details of the RNA extraction protocol and the description of NGS and GSEA analysis are provided in the Supplementary Materials.

### 2.6. Immunostaining and Histology

The ECTCs were fixed with 4% paraformaldehyde in PBS buffer for 15 min at room temperature, washed with PBS, and submitted to a core lab for embedding in paraffin blocks. The paraffin-embedded ECTCs were sliced into 5 µm sections, which were subjected to heat-induced epitope retrieval in sodium citrate buffer (10 mM, pH 6.0) at 97 °C for 5 min. After a brief PBS wash, the slices were blocked in 10% BSA (Sigma-Aldrich, St. Louis, MO, USA) in PBS, and then incubated with primary antibodies overnight at 4 °C; incubation with secondary antibodies was for 2 h at room temperature. Antibodies utilized include mouse monoclonal antibodies against the cardiac troponin T (MA5-12960, cTnT, 1:500, ThermoFisher Scientific, Waltham, MA, USA), collagen I (ab90395, COL-1, 1:200, Abcam, Waltham, MA, USA), and a rabbit polyclonal anti-connexin-43 antibody (sc-9059, H-150, 1:100, Santa Cruz Biotechnology, Dallas, TX, USA). The secondary antibodies were donkey anti-mouse Alexa Fluor^TM^ 568 and donkey anti-rabbit Alexa Fluor^TM^ 488 conjugated antibodies (A10037, A21206, 1:500, ThermoFisher Scientific, Waltham, MA, USA). Nuclear staining was done with DAPI (DAPI Fluoromount-G, Southern Biotech, Birmingham, AL, USA). A Zeiss LSM880 confocal microscope (Carl Zeiss Microscopy, White Plains, NY, USA) was utilized for fluorescence imaging of the ECTC slices.

### 2.7. Descriptive Statistics

Statistical analysis was performed using Microsoft Excel 2021 (Microsoft Corporation, Redmond, WA, USA) and OriginPro SR1 2022 (OriginLab Corp, Northampton, MA, USA). Group data are presented as mean ± SE. Statistical significance was evaluated with unpaired and paired Student’s *t*-test. Differences were considered significant at *p* < 0.05. Regression analysis was utilized to calibrate the flexible probe.

## 3. Results

### 3.1. Growth and Characterization of ECTCs

To create the ECTCs, we utilized a Matrigel^TM^/fibrinogen-based hydrogel. Fibrin is a biodegradable polymer, and fibrin-based constructs undergo extensive matrix remodeling as cells degrade fibrin, stimulating collagen deposition and the formation of an extracellular matrix [51]. Fibrin has been effectively employed to grow cardiac constructs using human embryonic stem cells [52] and neonatal cardiac cells [31,53]. During casting, we uniformly distributed hiPSCs into the molds at a final density of 10^7^ cells/mL. Over the next two days, hiPSCs proliferated, produced microcolonies, and remodeled the matrix, inducing gel condensation and the formation of an hiPSC fiber. The following CM differentiation occurred under tension within the fiber, which was attached to anchoring wires (Figure 1 and Figure S1). We observed initial spontaneous beating on days 13–15 of culturing. The early contractions were often asynchronous but became stronger and synchronic over time.

**Figure 2:**
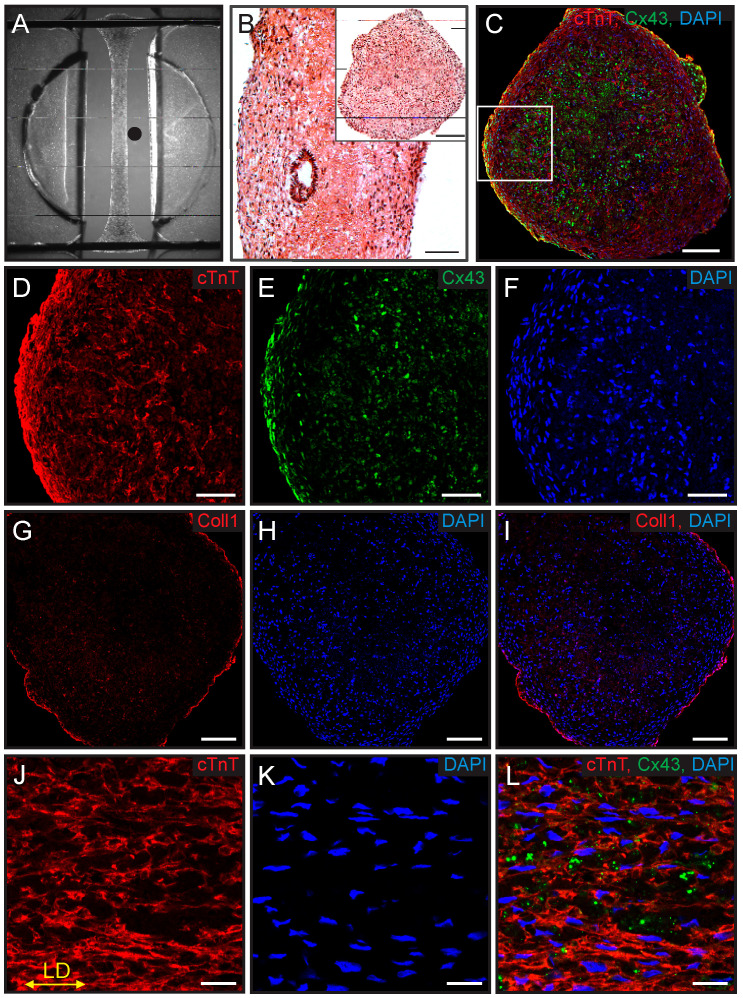
Histological imaging of ECTC on 45th day of differentiation. (**A**) ECTC in PDMS device located in the middle of the channel. The black dot on the right side of ECTC represents the position of the flexible probe. (**B**) H&E staining of longitudinal section with insert of cross-section. (**C**–**F**) Immunofluorescence of ECTC cross-section stained against cardiac Troponin T (cTnT, red), connexin-43 (C × 43, green), and DAPI (blue). (**G**–**I**) Images of the staining against collagen I. Scale bar in (**B**,**C**,**G**–**I**) is 100 µm, and in (**D**–**F**) is 20 µm. (**J**–**L**) Higher-magnification immunofluorescence images of the longitudinal sections of ECTC for cTnT and connexin-43. Scale bar is 20 µm. Yellow arrow points in the longitudinal direction of the construct.

A histological analysis of the ECTCs on the 45th day of culturing, shown in Figure 2, confirmed the differentiation of hiPSCs into cardiomyocytes. Figure 2A shows the uniformity of the construct diameter along its length, with a spreading at each end where the ECTC surrounds the supporting wires. Figure 2B shows hematoxylin and eosin (H&E) staining of longitudinal and cross (inset) sections of the ECTC and demonstrates a uniform cell distribution in both cross-sections. The ECTC is also characterized by connexin-43 positive gap junctions evenly allocated over the tissue (Figure 2E). The collagen deposition on the edge of the cardiac tissue fiber is very prominent in Figure 2G,I. The higher magnification of the longitudinal section stained against CM marker Troponin T (cTnT) in Figure 2J–L shows highly packed CMs mostly oriented horizontally along the ECTC (yellow arrow in Figure 2J). Additional immunofluorescent images demonstrating the ECTC structure are presented in Figure S4.

**Figure 3:**
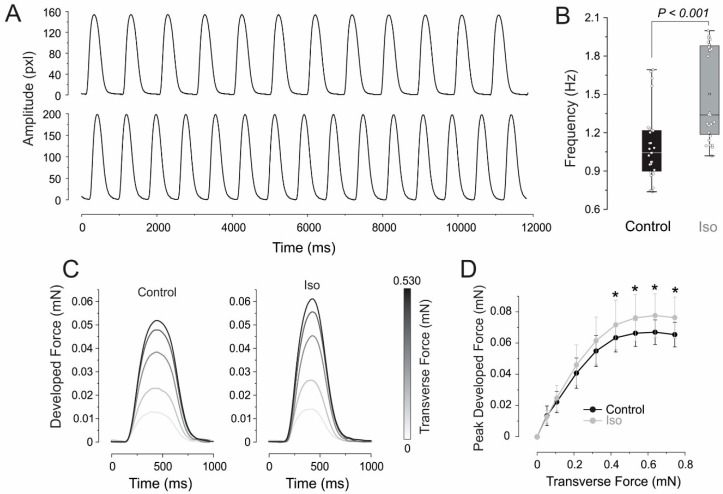
β-adrenergic stimulation in CC3 ECTC. (**A**) Representative uncalibrated contraction traces in control (upper) and at 1 µM isoproterenol. (**B**) Chronotropic effect of isoproterenol. (**C**) Developed force during control and isoproterenol application. (**D**) Effect of isoproterenol on Frank–Starling force–tension relationship, * *p* < 0.05, *N* = 8 different engineered cardiac tissue constructs (ECTCs).

To assess the ability of the ECTCs to respond to β-adrenergic stimulation, the constructs were incubated with 1 µM isoproterenol (I6504, Sigma-Aldrich, St. Louis, MO, USA) for two hours. The application of isoproterenol (Figure 3) caused a significant increase in the peak developed force at an applied transverse force greater than 0.318 mN (Figure 3C) and accelerated the spontaneous beating rate from 1.09 ± 0.27 Hz to 1.5 ± 0.36 Hz (Figure 3B) at an applied transverse force corresponding to the plateau in the force–tension curve in Figure 3D (* p < 0.05, *N* = 8 ECTCs).

### 3.2. Inotropic and Chronotropic Effects of Rapamycin in CC3 and TSC Constructs

**Figure 4:**
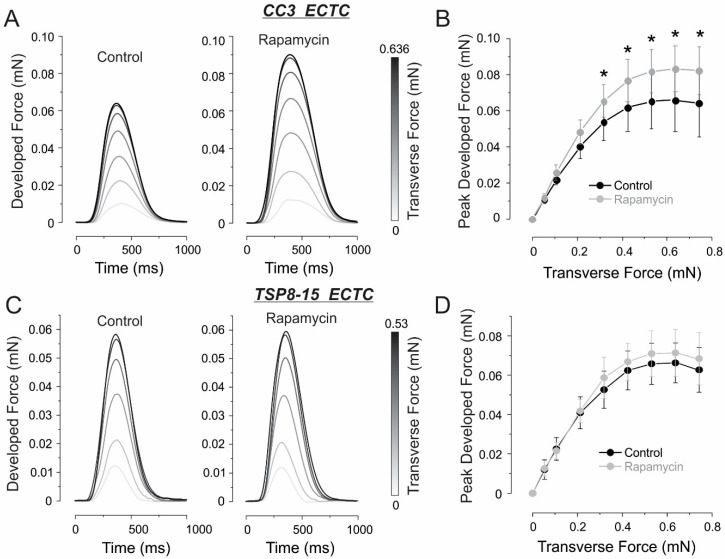
Inotropic response of the ECTC to treatment with 10 nM rapamycin. (**A**,**C**) Representative superimposed developed force traces as a function of applied transverse force in control (left) and after rapamycin application (right) are shown for CC3 (**A**) and TSP8-15 (**C**) cardiac constructs. (**B**,**D**) Frank–Starling force–tension relationship for CC3 (**B**), *N* = 9 ECTCs and TSP8-15 (**D**), *N* = 8 ECTCs. Values are means ± SD. * *p <* 0.05.

**Figure 5:**
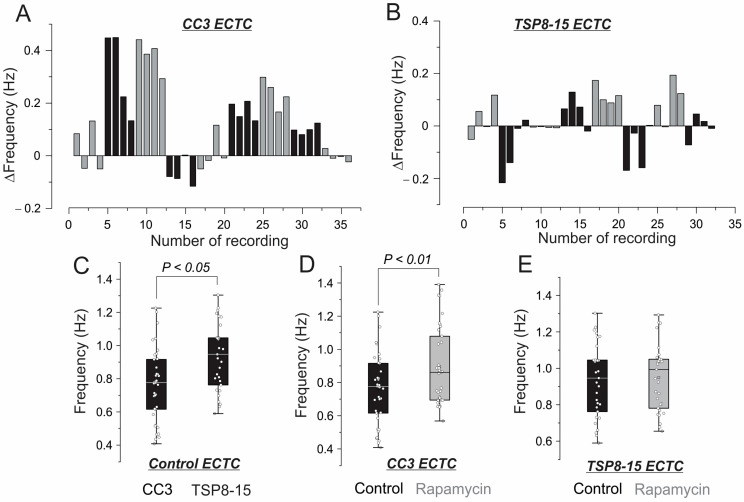
Chronotropic response of the CC3 and TSP8-15 ECTCs to application of 10 nM rapamycin. Difference in frequency of spontaneous beating rate before and after treatment with rapamycin for CC3 ECTC (**A**) and TSP8-15 (**B**). (**C**) Faster beating rate in control TSP8-15. (**D**) Increase in contraction frequency by rapamycin in CC3 cardiac constructs (*p* < 0.01, *N* = 36 recordings), and (**E**) no significant alteration in TSP8-15 constructs (*N* = 32 recordings). Values are means ± SD. In total, 9 CC3 and 8 TSP8-15 ECTCs were analyzed. Different ECTCs are marked with gray and black in (**A**,**B**).

Figure 4 shows the effects of rapamycin on contractility in the CC3 and TSP8-15 ECTCs. Treatment of the CC3 constructs with 10 nM rapamycin for two days caused a significant increase in developed force amplitude, from 21.6% to 28.2% for applied tensions from 0.32 mN to 0.74 mN, respectively (Figure 4A,B: * p < 0.05, *N* = 9 ECTCs). The application of rapamycin in the TSP8-15 constructs caused an insignificant elevation of contractility (Figure 4C,D: *N* = 8 ECTCs). The change in the developed force in response to rapamycin in both types of ECTCs as a function of stretch is demonstrated in Figure S5. Figure 5 represents the change in the frequency of spontaneous beating rate following rapamycin treatment. In each ECTC, we acquired and analyzed four recordings obtained at a gradually increased lateral tension of 0.424, 0.53, 0.636, and 0.742 mN, which correspond to the plateau phases in Figure 3D and Figure 4D,E. After the application of rapamycin, the recordings were repeated under the same conditions. Spontaneous beating rates were significantly higher in the TSP8-15 constructs (0.93 ± 0.2 Hz, *N* = *32* recordings) than in the CC3 constructs (0.76 ± 0.22 Hz, *N* = *36* recordings) (Figure 5C, * p < 0.05). In response to rapamycin, we observed a significant beating acceleration in the CC3 ECTCs from 0.76 ± 0.22 Hz to 0.89 ± 0.24 Hz (Figure 5D: * p < 0.05, *N* = 36 recordings). It should be noted that the CC3 cardiac constructs had heterogeneous chronotropic sensitivity to rapamycin. In particular, one construct exhibited a slightly negative effect of rapamycin and three did not show any effect of rapamycin, whereas five others demonstrated substantial acceleration (Figure 5A). We did not find a considerable impact of rapamycin on the beating rate in the TSP8-15 ECTCs (Figure 5B–E).

### 3.3. Elastic Properties

The calculation of elastic modulus was based on the ECTC stretch–stress diagram, which is illustrated in Figure S6. The slope of the linear part of the stretch–stress curves was utilized to calculate Young’s modulus (Figure S6B,C,E,F). The value of the elastic modulus was slightly lower in the CC3 ECTCs, 5.4 ± 1.7 kPa, than in the TSP8-15 ECTCs, 6.8 ± 1.8 kPa (Figure S6G). The application of rapamycin did not reveal significant changes in elasticity in either the CC3 (5.7 ± 2.1 kPa) or TSP8-15 (6.7 ± 1.7 kPa) ECTCs (Figure S6H,I).

### 3.4. Next-Generation Sequencing (NGS) and Gene Ontology Enrichment Analysis of Differentially Expressed Genes (DEGs)

**Figure 6:**
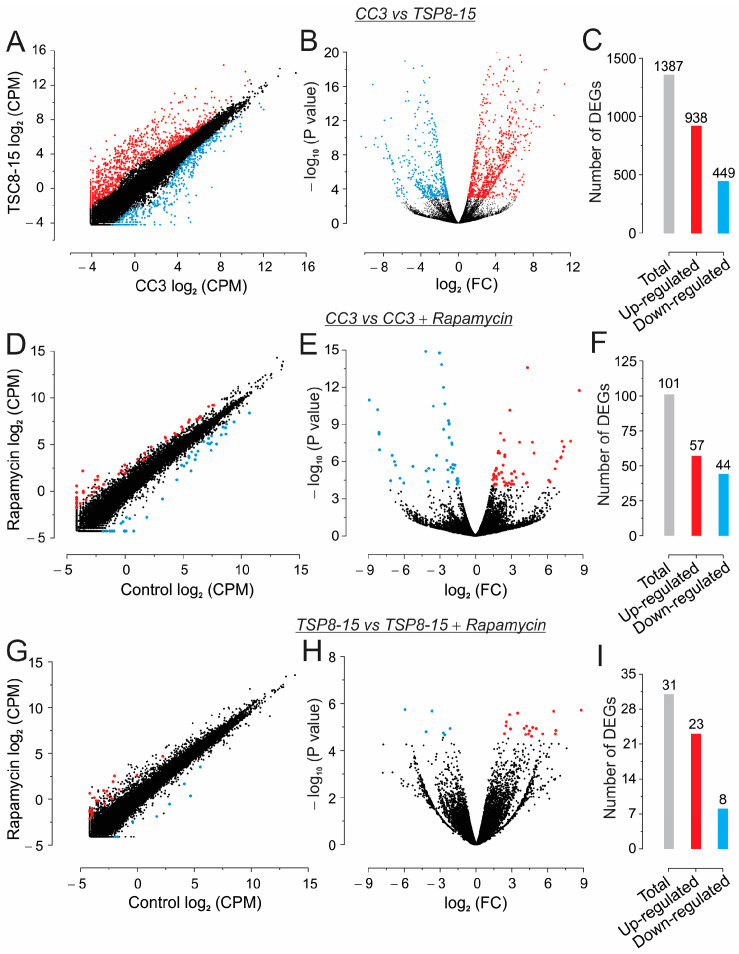
Scattered (left) and volcano (right) plots of RNA sequencing data. Transcriptomic comparison in CC3 vs. TSP8-15 ECTCs (**A**–**C**), CC3 vs. CC3 and rapamycin (**D**–**F**), and TSP8-15 vs. TSP8-15 and rapamycin (**G**–**I**). Red dots indicate up-regulated and blue dots down-regulated genes. Black dots indicate not DEGs. CPM, count per million. FC, fold change. False discovery rate (*FDR*) < 0.05.

To characterize the gene expression profiles in the CC3 and TSP8-15 constructs, we conducted an NGS analysis. In total, when comparing CC3 vs. TSP8-15, 1387 DEGs were identified, including 938 up-regulated and 449 down-regulated DEGs (Figure 6A–C, Figures S7 and S8, and Tables S1 and S2). We also looked for DEGs in response to treatment with 10 nM rapamycin in both the CC3 and TSP8-15 ECTCs. A comparison of the vehicle-treated and rapamycin-treated constructs revealed 101 DEGs with 57 up- and 44 down-regulated genes in the CC3 ECTCs (Figure S9 and Table S3) and 31 DEGs with 23 and 8 of up- and down-regulated genes, respectively, in the TSP8-15 constructs (Figure S10 and Table S4).

**Figure 7:**
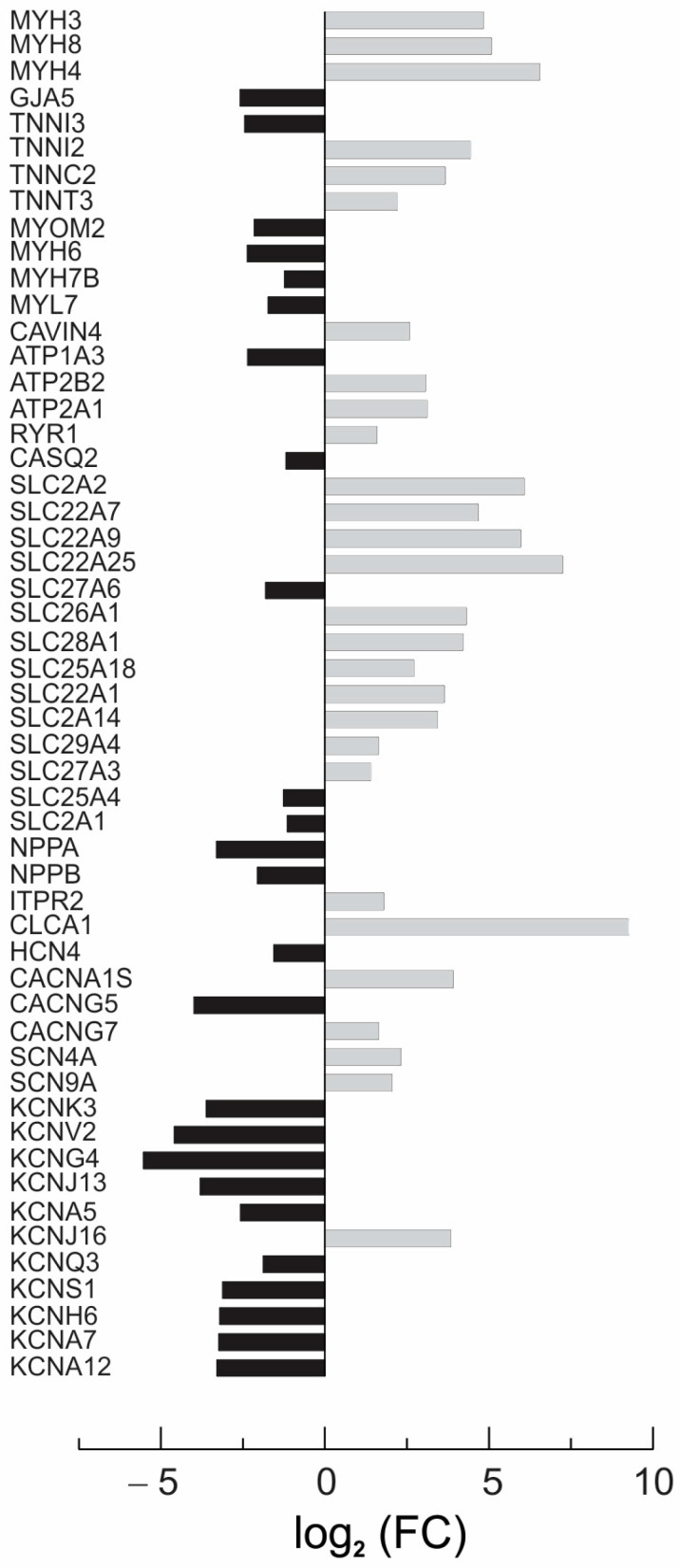
Fold change (FC) of cardiac-specific TSP8-15 genes that are differentially expressed relative to those in CC3.

Figure 7 presents an analysis of the activity of cardiac-specific DEGs associated with action potential generation, calcium signaling, and contraction (Figure S11 and Table S5). Relative to the CC3 constructs, we observed a down-regulation of the potassium channel KCNK3, KCNV2, KCNG4, KCNJ13, KCNA5, KCNQ3, KCNS1, KCNH6, KCNA7, and KCNK12 genes in the TSP8-15 constructs. In addition, two genes that code sodium channels, SCN4A and SCN9A, were up-regulated relative to CC3 tissue. There was alternation in the expression of the genes involved in calcium handling (RYR1, CASQ2, ATP2B2, and ATP2A1), and in gene ATP1A3, which codes for one of the subunits of the sodium pump. In addition, we detected differences in the expression of genes that control contractile function, such as MYH6, MYH7B, TNNI3, TNNI2, TNNC2, TNNT3, MYH3, MYH8, MYH4, and MYL7, and genes encoding transmembrane transporters (SLC27A6, SLC26A1, SLC28A1, SLC25A18, SLC22A1, and SLC2A14) between the CC3 and TSP8-15 constructs. Supplementary Tables S1–S5 provide a complete list of up- and down-regulated cardiac-specific DEGs and a list of the 100 top-scored DEGs detected in the TSP8-15 ECTCs when compared to their CC3 counterparts. Supplementary Tables S4 and S5 list the genes differentially expressed in response to rapamycin in the CC3 and TSP8-15 ECTCs. Rapamycin exposure did not result in the differential expression of cardiac-specific genes in either CC3 or TSP8-15 associated with rapamycin treatment.

We performed a gene ontology (GO) functional enrichment analysis for the DEG sets to identify significantly enriched biological processes, cellular components, and molecular functions resulting from a comparison of the CC3 and TSP8-15 ECTC constructs as affected by rapamycin treatment. [54].

**Figure 8:**
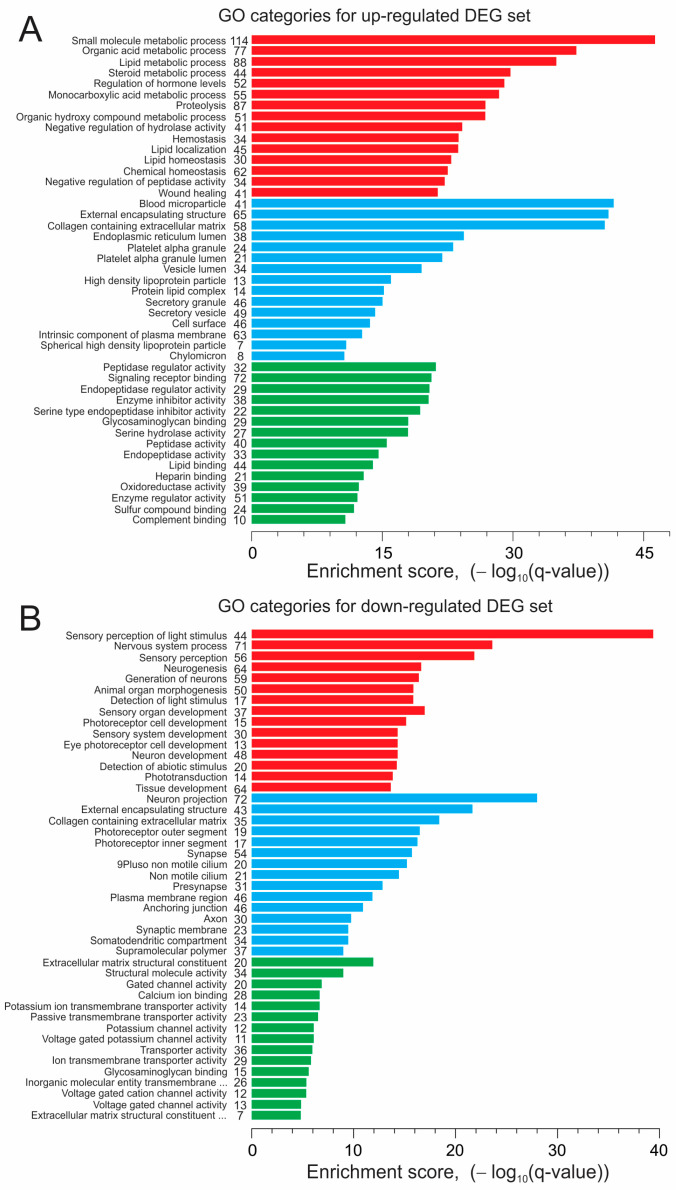
Gene ontology enrichment analysis of CC3 vs. TSP8-15 ECTCs, up- (**A**) and down-regulated (**B**) DEG sets. The top 15 GO terms for biological processes (red), cellular components (blue), and molecular function (green) are shown. The numbers in the column are the counts of genes in overlap.

Figure 8 presents the top fifteen GO terms in each of three categories, ranked by the enrichment score attained during the analysis of the DEG set that resulted from the comparison of CC3 and TSP8-15 gene profiles. The top-ranked biological processes for up-regulated gene sets are related to the small-molecule regulation of metabolism, pathways involving organic acid metabolism, metabolism of lipids, and monocarboxylic acids, and to reducing the rate of the hydrolysis of peptides. The related top-ranked cellular components are blood microparticle, external encapsulating structure, collagen containing extracellular matrix, and others. The highest scored molecular functions are peptidase regulator activity, signaling receptor binding, endopeptidase regulator activity, enzyme regulator activity, and others. The down-regulated genes are involved in biological processes related to neurogenesis, sensory perception, the development of neurons, photoreceptor cells, the sensory system, phototransduction, and tissue development. The highest scored terms for cellular components are neuron projection, external incapsulating structure, collagen containing extracellular matrix, and photoreceptor outer segment. The top-ranked terms for molecular function are extracellular matrix structural constituent, structural molecular activity, gated channel activity, calcium ion binding, potassium ion transmembrane transporter activity, and others. The genes associated with annotations in each category are illustrated in overlap matrix maps (Figures S7 and S8).

**Figure 9:**
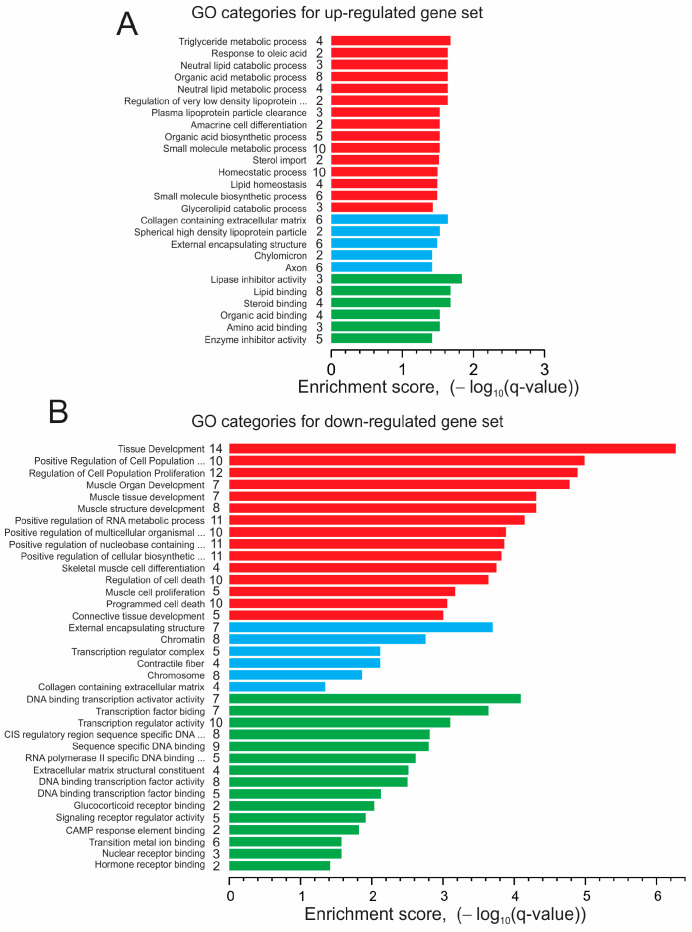
Gene ontology enrichment analysis of up- (**A**) and down-regulated (**B**) gene sets. Genes were differentially expressed in response to rapamycin treatment of CC3 ECTCs. Bar charts show the top 15 GO annotations and terms for biological processes (red), cellular components (blue), and molecular function (green). The numbers in the column are the counts of genes in overlap.

The data of the GO analysis of the DEG sets obtained in response to the treatment of the CC3 ECTCs with rapamycin are shown in Figure 9A,B. The top-ranked terms and annotations in biological processes, cellular components, and molecular function in the up-regulated gene set were mostly related to the metabolism and catabolism of triglycerides, fatty acids, and lipids, and comprise the triglyceride metabolic process, response to oleic acid, neutral lipid catabolic process, organic acid metabolic process, neutral lipid metabolic process, regulation of very low-density lipoprotein particle remodeling, plasma lipoprotein particle clearance, lipase inhibitor activity, lipid binding, steroid binding, organic acid binding, amino acid binding, and other processes.

With respect to down-regulated DEGs, the top-ranked annotations and terms are related to tissue development, cell cycle, and proliferation; in particular, the positive regulation of cell population proliferation, regulation of cell population proliferation, muscle organ development, muscle tissue development, muscle structure development, positive regulation of the RNA metabolic process, DNA binding transcription activator activity, transcription factor binding, transcription regulator activity, cis regulatory region sequence-specific DNA binding, sequence-specific DNA binding, RNA-polymerase-II-specific DNA binding, transcription factor binding, and so on. The corresponding overlap matrix maps are shown in Figures S12 and S13. We did not find any annotations or terms to be significantly enriched when analyzing genes differentially expressed in the TSP8-15 ECTCs in response to rapamycin.

## 4. Discussion

### 4.1. Growing ECTCs

To create cardiac constructs, we utilized a novel approach of encapsulating hiPSCs into a Matrigel^TM^/fibrinogen-based matrix with the following differentiation of CMs taking place directly within the scaffold. Typically, growing ECTCs requires two main steps: the differentiation of CMs from hiPSCs and casting the CM–scaffold mixture in molds to undergo 3D assembly. The combination of two procedures in one course avoids the dissociation of previously differentiated CMs, which disrupts the electrical and mechanical cell-to-cell interface, and allows the cardiac tissue construct to develop more naturally. To date, approaches wherein hiPSCs are encapsulated and differentiated within a soft scaffold are not widespread. There are relatively few studies involving the construction of skeletal muscle [55] and cardiac microtissues [56,57]. Kerscher et al. have used a hybrid biomaterial poly(ethylene glycol)-fibrinogen hydrogel [56] and degradable hydrogel gelatin methacryloyl [57] to successfully encapsulate and differentiate hiPSCs. In that study, a micro-island cardiac tissue from single encapsulated hiPSCs began spontaneously contracting between days 9–11 and developed mature structural features over time [56]. The 3D environment was also implemented to differentiate CMs from hiPSCs and human embryonic stem cells (hESCs) using stirred suspension bioreactors in which CMs differentiated as cell aggregates into spheroids containing a high fraction of cardiomyocytes [58,59,60].

It is known that CM differentiation is a multifaceted and spatially and temporally regulated process. Multiple environmental and biochemical factors have been demonstrated to influence the efficiency of CM differentiation. Among them are the regulation of the time of activation and blocking of Wnt, FGF, and TGF signaling pathways, cell density, cell culture matrix, heparin, and insulin [43,61,62]. It is important to note that in contrast to 2D differentiation in monolayers, differentiation in the 3D environment is initiated after hiPSCs have formed microspheres. In our experiments, hiPSCs are allowed to proliferate for two days to develop 3D microcolonies inside a Matrigel^TM^/fibrinogen-based matrix.

The hiPSC density is essential for efficient differentiation. Typically, it is recommended to start differentiation at >80% confluency, which should be reached in two to three days [63]. It has been demonstrated that the treatment of hESCs with bone morphogenic protein BMP4 initiates differentiation on the outer edge of the hESC colonies, suggesting that an increase in the number of small colonies advances CM differentiation efficiency [64]. These data support the view that the cell cycle is a critical factor controlling the process of differentiation. Previous reports have shown that glycogen synthase kinase-3 beta (GSK3β) regulates the cell cycle via cyclin D1/E [65] and the chromatin alignment of mitotic cells [66]. Therefore, in the first stage of cardiac differentiation, when CHIR99021 is applied to inhibit GSK3β, mesoderm development is significantly influenced by the metabolic state of the hiPSCs, which in turn is associated with cell cycle phases. In our approach, we initiated the cardiac differentiation two days after hiPSC mixture molding, when the hiPSCs proliferated and formed multiple microcolonies. We hypothesize that under these conditions, the encapsulated population of stem cells is metabolically more homogeneous and hence more prone to successful differentiation.

The extracellular matrix is also fundamentally involved in cardiac development. A fibrin-based scaffold can facilitate the differentiation of adipose-derived stem cells toward cardiac cells [67], enhance the differentiation of neurons from neural progenitor cells [68,69], and promote osteogenesis [70]. As discussed above, we utilized a fibrin-based scaffold enriched with Matrigel^TM^ to prepare the hiPSC mixture. The ability of Matrigel^TM^ to promote cardiac differentiation has previously been demonstrated by means of the matrix sandwich method [71].

### 4.2. Inotropic Effect of Rapamycin in CC3 ECTCs

mTOR signaling is quite complex and regulates cardiac functionality on the transcriptional and translational levels and on the level of direct interaction with regulatory molecules to elicit an immediate effect on cardiac contractility. Mollmann et al. reported a reversible concentration-dependent negative inotropic effect when 10^−9^–10^−5^ M rapamycin was added for 15 min to human cardiomyocytes enzymatically isolated from right atrial appendages [72]. This effect was reversible. They also observed a rapamycin-induced decrease in diastolic cell length with a maximal value of 19.8 ± 2.5% at 10^−5^ M drug. The authors suggested that the interaction of Ryanodine Receptor (RyR) with rapamycin and FK506-binding protein FKBP12, which plays an essential role in regulation of RyR, could destabilize RyR and result in increased calcium leakage from the sarcoplasmic reticulum [72,73]. In rat cardiomyocytes isolated from the left ventricle, the application of 20 µM rapamycin for 8 h significantly increased Akt phosphorylation, inhibited the phosphorylation of mTOR, but did not significantly change either the peak shortening or the maximal contraction/relaxation velocity of shortening [74].

In our study we observed a substantial increase in ECTC contractility in the CC3 cardiac constructs after two days of treatment with 10 nM rapamycin. There are several possible explanations for this difference. It is known that atrial and ventricular cardiomyocytes differ in structure and Ca^2+^ signaling. One of the key structural differences is the lack of T-tubules in atrial myocytes [75], resulting in two distinct populations of RyRs, junctional and non-junctional [76], which result in a more complex mechanism of excitation-contraction coupling [77]. In addition, three RyR isoforms have been described, and although RyR isoforms have about 65% homology, the different RyR isoforms respond differently to some molecule modulators [78]. There is evidence that mTOR can affect Ca^2+^ signaling via the modulation of the inositol trisphosphate pathway (IP3) [79]. In vascular smooth muscle cells, rapamycin disrupts the FKBP and IP3 receptor association, resulting in an increase in the amplitude of the caffeine-evoked [Ca^2+^]_i_ rise [80].

Another mechanism that potentially could be involved in the inotropic effect of rapamycin in the CC3 ECTCs is glucose metabolism. In mTOR downstream signaling, p70S6K is a key kinase in the negative feedback to regulate the insulin signaling cascade [81]. It has been proposed that analogs of rapamycin—rapalogs—can relieve the negative feedback inhibition of Akt by suppressing p70S6K. The inhibition of mTOR with rapamycin increases upstream Akt phosphorylation [82], which phosphorylates target proteins affecting glucose uptake and metabolism [83]. In work by Sen et al., the pretreatment of rats with rapamycin for 7 days increased cardiac power when the rat hearts were subjected to increased workload, but no change in oxygen consumption or in mitochondrial efficiency was detected. They found that rapamycin significantly reduces workload-induced mismatch between glucose uptake and heart oxidative capacity and thereby can preclude metabolic remodeling and load-induced contractile disfunction [84].

### 4.3. Resistance to Rapamycin in TSP8-15 ECTCs

In our work we observed significant inotropic and chronotropic effects of the rapamycin in the CC3 ECTCs, but no changes were detected in the TSP8-15 tuberous sclerosis ECTCs. The TSP8-15 cell lineage has a heterozygous loss-of-function missense mutation in the *TSC2* gene. Mutations in *TSC1*/*TSC2* genes are often associated with cardiac rhabdomyomas in TSC. Resistance to rapamycin is commonly attributed to its inability to inhibit cell proliferation [85]. Several mechanisms have been proposed for rapalog resistance, and it has been found that the translational regulators S6Ks and 4E-BP1 are not equally impacted by treatment of rapamycin [86], which could be caused by a structural alternation or post-translational modifications of mTORC1.

It was also suggested that mTORC1 substrates could have a different binding affinity, so that only minor mTORC1 structural alterations could lead to the inhibition of one substrate, but could be much less effective with respect to another substrate [87]. There are also data that mTORC1 can be regulated by the phosphorylation of raptor [88]. In renal cell carcinoma, the cell lineage glycogen synthase kinase-3β can directly phosphorylate 4EBP1 and activate the mTORC1 downstream signaling cascades [89]. In work by Kang et al., the insensitivity of certain mTORC1 substrates to phosphorylation is accounted for by the sequence composition of an mTORC1 phosphorylation site, especially by the presence of serine or threonine as the phosphoacceptor [90]. The authors believe that “substrate quality” is a key factor of substrate sensitivity to modulators of the pathway. Recently, it has been demonstrated that the mTOR3 complex can contribute to rapamycin resistance [91]. Specifically, the ETS transcription factor ETV7 interacts with mTOR to assemble a complex, which lacks critical mTORC1/mTORC2 components but exhibits bimodal mTORC1/mTORC2 activity. This mechanism of resistance to rapamycin was suggested to explain tumorigenicity in a rhabdomyosarcoma mouse model and attributed to malignant cancer [92]. Other mechanisms responsible for rapamycin resistance may be low levels of activation of Akt and mutations in FKBP12, which prevent the formation of the FKBP12–rapamycin complex [93,94].

Given the loss-of-function mutation of the *TSC2* gene that encodes tuberin, it is worthwhile to consider the hamartin–tuberin interaction. Hamartin is a product of the *TSC1* gene, and the hamartin–tuberin interaction is mediated by coiled-coil domains [95]. Tuberin can act as a chaperone, preventing hamartin self-aggregation to keep the tuberin–hamartin complex in soluble form [96]. The inactivation of mTORC1 requires the translocation of the *TSC1*/*TSC2* complex to the lysosome membrane [97]. To process downstream signaling and regulate mTORC1 activity, tuberin has a domain homologous to the GTP-activating protein, which is responsible for Rheb-GTP hydrolysis, converting it to Rheb-GDP [98]. Since a significant effect of rapamycin on contraction force and contraction rate was detected only in healthy ECTC counterparts, it can be hypothesized that the hamartin–tuberin complex plays a role in the binding of FKBP12–rapamycin to mTORC1, and that the structural changes induced in mTORC1 due to Rheb-GTP dephosphorylation could affect the binding affinity of mTORC1 to the FKBP12–rapamycin complex.

### 4.4. Effect of Rapamycin on CC3 ECTC Gene Expression

The gene ontology enrichment analysis for the up-regulated gene set indicated significant annotations and terms related to fatty acid/lipid metabolism (Figure 9A, Figures S9 and S12, and Tables S3 and S6). For example, APOC3 is particularly important as a regulator of the transport and metabolism of triglyceride in cells [99,100]. Diacylglycerol acyltransferase 2 (DGAT2) catalyzes the reaction in the synthesis of triglycerides. Apolipoprotein-A2 (APOA2) is the second most common protein in high-density lipoproteins and plays a key role in triglyceride catabolism by regulating lipoprotein lipase activity [101]. Fatty acids can activate multiple intracellular signaling pathways that are related or unrelated to gene transcription modulation [102]. It has been demonstrated that in rat hepatocytes, rapamycin affects lipid metabolism by increasing the rate of oxidation of saturated and unsaturated fatty acids [103] and inhibits glucose metabolism in human cells [104,105], but the interpretation of these findings in the context of our study is difficult because of the obvious differences between cardiomyocytes and hepatocytes, and the wide range of different rapamycin concentrations used for studies of isolated cells, cellular monolayers, and ECTCs. In hiPSC-derived cardiomyocytes, fatty acids improve contractile force, increase action potential upstroke velocity, and enhance oxidative capacity [106]. Since mTOR1 stimulates protein synthesis [92], rapamycin also has an effect on the metabolism of amino acids. In our study, the genes AGXT, GATM, PAH, and TDO2 were enriched in the GO annotations: organic acid metabolic process and small-molecule metabolic process (Figure S9 and Table S6). The products of these genes are serine–pyruvate aminotransferase, arginine–glycine amidinotransferase, phenylalanine hydroxylase, and tryptophan 2,3-dioxygenase. These enzymes are involved in glycine, serine, arginine, threonine, and tryptophan metabolism [107,108], and these metabolic changes could improve contractility.

In our gene ontology enrichment analysis of down-regulated DEGs, the top-ranked biological processes, cellular components, and molecular functions were mostly associated with tissue, muscle, and organ development, as well as cell proliferation (Figure 9B, Figures S9 and S13, and Tables S3 and S7). For instance, the genes EGR1, EGR3, and EGR4 belong to the early growth response (EGR) family of genes, which dynamically modulates gene expression involved in cell growth and differentiation in response to a variety of cell stimuli. In particular, EGR1 has many transcriptional targets and also regulates multiple tumor suppressors, including TGFβ1, PTEN, and p53 [109,110,111]. The genes NR4A3 and NR4A1 are related to the NR4A subfamily encoding nuclear hormone receptors acting as transcriptional activators. These gene products are implicated in Ca^2+^ homeostasis and in different cardiac pathologies, such as post-myocardial infarction remodeling, ischemia–reperfusion injury, ventricular hypertrophy, and atrial fibrillation [112,113,114]. A cAMP-dependent transcriptional factor, ATF3, is essential for the regulation of cellular adaptive response, and its expression is highly elevated following various cardiac stresses. The expression of ATF3 in cardiomyocytes can result in hypertrophy, fibrosis, and cardiac dysfunction [115,116]. The XIRP2 product supports cytoskeleton actin filament binding and has a critical role in cardiac conduction [117]. The cardiac ankyrin repeat domain 1 protein ANKRD1 is characterized as a cardio-enriched transcriptional co-factor and stress-inducible myofibrillar protein [118]. It may also be involved in the myofibrillar stretch-sensor system [119].

## 5. Conclusions

We implemented our novel approach of direct CM differentiation to create the I-Wire cardiac construct model of TSC disease and investigated the effect of the mTOR inhibitor rapamycin on contractility in healthy CC3 and TSC patient-derived TSP8-15 ECTCs. Direct CM differentiation imitates in vivo cardiac development and, as with many microphysiological models, better recapitulates cardiac tissue properties than monolayer culture on plastic [1]. Rapamycin revealed positive inotropy and chronotropy, i.e., increased contractility and heart rate, in healthy CC3 ECTCs and did not have a significant effect in the TSP8-15 ECTCs. When comparing cardiac-specific DEGs in the TSP8-15 ECTCs relative to their healthy counterparts, we found that ten genes encoding potassium channels, two genes encoding sodium channels, four genes involved in calcium handling, ten genes controlling contractile function, and six genes encoding transmembrane transporters were altered. Rapamycin induced the differential expression of 101 genes in the CC3 ECTCs that encompassed 57 up- and 44 down-regulated genes, and 31 genes in the TSP8-15 ECTCs with 23 and 8 up- and down-regulated genes, respectively. The GO analysis showed that the up-regulated genes in the CC3 ECTCs were significantly enriched in processes involved in metabolism of lipids and fatty and amino acids, while the down-regulated genes were mostly associated with cell proliferation and muscle and tissue development. We hypothesized that the hamartin/tuberin complex is involved in the binding of FKBP12–rapamycin to mTORC1.

## 6. Limitations

It is known that cardiomyocyte differentiation outcomes can be highly changeable, and any protocols consistently produce a mixed population of cardiomyocytes and non-cardiomyocyte cells, including smooth muscle cells, endothelial cells, fibroblasts, and undifferentiated iPSCs in varied proportions [47,120]. In addition, differentiation protocols yield a mixed population of subtypes of cardiomyocytes, which include ventricular, atrial, and pacemaker-like cells [120,121]. Obviously, the force of contraction depends on the number and manner of arrangement of cardiomyocytes and other cells in cardiac tissue. The presence of different cell types complicates the interpretation of the data obtained from the ECTC. In our work, based on the calibration of the flexible sensor, we were able to express the relative values of ECTC contraction in Newtons, which is important for the subsequent comparison of our data with measurements obtained using other measuring systems. It should be noted that cardiomyocytes in vivo constitute 30–40% of the total cardiac cells, occupying about 70–80% of the heart volume [122,123]. Both cardiomyocytes and non-cardiomyocyte cells respond to physiological stress and pathophysiological remodeling of the heart, and the pathology can progress through the interactions between different cardiac cell types [120,122]. This indicates the importance and necessity of co-culturing different types of cells in disease modeling. However, a full cellular characterization of the ECTCs is beyond the scope of this preliminary, explorative project.

Another limitation that has been widely described in the literature is the lack of maturity of the cardiomyocytes differentiated from iPSCs [124,125]. The presence of structural and functional immaturity means that the translation of findings from pharmacological interventions is not straightforward [125,126]. Although direct differentiation in the 3D matrix better recapitulates the physiological environment than CM differentiation on 2D plates, it is impossible to completely recapitulate the embryonic environment during heart development. However, the presence of disease-associated phenotypes in immature cells supports the idea that pathology may develop before the onset of clinical symptoms. This makes hiPSC-based models useful not only for studying disease mechanisms but also for targeting the presymptomatic phase of the disease [127].

As is known, cardiac rhabdomyoma is a common manifestation of TSC and it is often accompanied with arrhythmias. It is generally accepted that anatomical and functional heterogeneities are closely involved in arrhythmogenesis, especially if they are associated with the conduction system. However, the question that remains unanswered is whether mutations in either the *TSC1* or *TSC2* genes can lead to abnormalities in action potential formation or impaired calcium homeostasis and signaling, which in turn trigger abnormal impulses in cardiomyocytes without structural distortions, while rhabdomyoma serves as an obstacle to break the wavefront and generate arrhythmic premature stimuli. The dimension of the ECTC is too small to support occurrence of the re-entrant arrhythmias; therefore, rhabdomyoma, if it arose in ECTCs, would not lead to tachycardias.

In our study we observed a significant change in the expression of cardiac-specific genes in the TSP8-15 ECTCs compared to the CC3 ECTCs. These are genes coding subunits of potassium and sodium channels, proteins involved in calcium homeostasis, and contractile proteins. These changes correlate with an increase in the contraction rate in the TSP8-15 ECTCs, suggesting that in response to certain physiological or pharmacological interventions, the TSC heart can be more prone to arrhythmia initiation than the normal heart.

## Data Availability

Data will be made available on request.

## Supplementary Materials

The following supporting information can be downloaded at https://www.mdpi.com/article/10.3390/bioengineering11030234/s1, Figure S1. Phase contrast images of hiPSCs encapsulated in Matrigel^TM^/fibrinogen-based matrix. Figure S2. Diagram of the registration system. Figure S3. Schematic representation of force vectors exerted in ECTCs. Figure S4. Histological imaging of ECTC on day 29. Figure S5. The change in the peak developed force (ΔFD) in response to rapamycin as a function of the stretch. Figure S6. Effect of rapamycin on elastic properties of CC3 and TSP8-15 ECTCs. Figure S7. Comparison of CC3 and TSP8-15 ECTCs. Overlap matrix of relationship between the top-scored GO terms and the corresponding up-regulated DEGs and their products. Figure S8. Comparison of CC3 and TSP8-15 ECTCs. Overlap matrix of relationship between the top-scored GO terms and the corresponding down-regulated DEGs and their products. Figure S9. Gene expression in CC3 ECTCs in response to rapamycin. Figure S10. Gene expression in TSC8-15 ECTCs in response to rapamycin. Figure S11. Cardiac-specific TSC8-15 ECTC genes differentially expressed relative to those in CC3. Figure S12. Comparison of CC3 and CC3/+rapamycin ECTCs. Overlap matrix of relationship between the top-scored GO terms and the corresponding up-regulated DEGs and their products. Figure S13. Comparison of CC3 and CC3/+rapamycin ECTCs. Overlap matrix of relationship between the top-scored GO terms and the corresponding down-regulated DEGs and their products. Table S1. Up-regulated top-ranked 100 DEGs obtained by comparison of CC3 and TSP8-15 ECTCs. Table S2. Down-regulated top-ranked 100 DEGs obtained by comparison of CC3 and TSP8-15 ECTCs. Table S3. DEGs detected in CC3 ECTCs in response to application of rapamycin. Table S4. DEGs detected in TSP8-15 ECTCs in response to application of rapamycin. Table S5. Heart-specific DEGs detected by comparing CC3 and TSP8-15 ECTC gene expression data sets. Table S6. Gene ontology categories significantly altered in up-regulated group of genes in response to application of rapamycin in CC3 ECTCs. Table S7. Gene ontology categories significantly altered in down-regulated group of genes in CC3 ECTCs. Video. Contracting cardiac tissue construct.
