# Supplementary material for: Contractile and Genetic Characterization of Cardiac Constructs Engineered from Human Induced Pluripotent Stem Cells: Modeling of Tuberous Sclerosis Complex and the Effects of Rapamycin
Source: Bioengineering (Basel). 2024 Feb 28;11(3):234. doi: 10.3390/bioengineering11030234 (PMC10968530; doi:10.3390/bioengineering11030234)
Supplement: Supplementary file 1 [file bioengineering-11-00234-s001.zip › bioengineering-2846441-supplementary/bioengineering-2846441-supplementary revised.pdf]

bioengineering

Supplementary Material

Contractile and Genetic Characterization of Cardiac Constructs Engineered from Human Induced Pluripotent Stem Cells: Modeling of Tuberous Sclerosis Complex and the Effects of Rapamycin

Veniamin Y. Sidorov [1,2,*], Tatiana N. Sidorova [3], Philip C. Samson [1,4], Ronald S. Reiserer [1,4], Clayton M. Britt [1,4], M. Diana Neely [5], Kevin C. Ess [5] and John P. Wikswo [1,2,4,6]

[1] Vanderbilt Institute for Integrative Biosystems Research and Education, Vanderbilt University, Nashville, TN, 37235, USA; philip.samson@vanderbilt.edu (P.C.S.); ron.reiserer@vanderbilt.edu (R.S.R.); clayton.britt@vanderbilt.edu (C.M.B.); john.p.wikswo@vanderbilt.edu (J.P.W.)
[2] Department of Biomedical Engineering, Vanderbilt University, Nashville, TN, 37235, USA
[3] Department of Anesthesiology, Vanderbilt University Medical Center, Nashville, TN, 37232, USA; tatiana.n.sidorova@vumc.org (T.N.S.)
[4] Department of Physics and Astronomy, Vanderbilt University, Nashville, TN, 37212, USA
[5] Department of Pediatrics, Vanderbilt University Medical Center, Nashville, TN, 37232, USA; diana.neely@vumc.org (M.D.N.); kevin.ess@vumc.org (K.C.E.)
[6] Department of Molecular Physiology and Biophysics, Vanderbilt University, Nashville, TN, 37232, USA
* Correspondence: v.sidorov@vanderbilt.edu (V.Y.S.)

We present supplementary figures and text in the order in which they are presented in the body of the manuscript, preceded by the heading of the section in which they are first referenced.

2.3. hiPSC differentiation

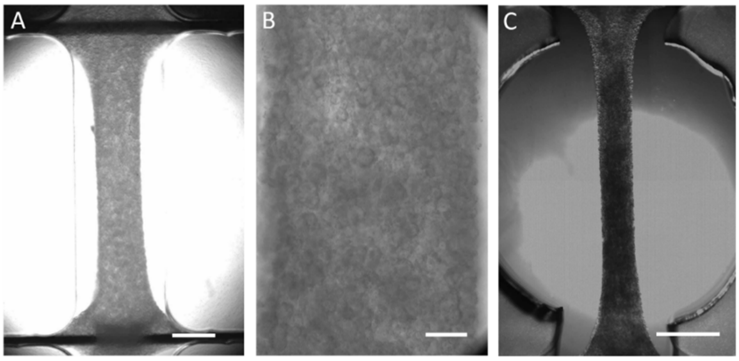

Figure S1: Phase contrast images of hiPSCs encapsulated in Matrigel™/fibrinogen-based matrix at low (**A**) and higher magnification (**B**) on day 0, before onset of the cardiomyocyte differentiation protocol. Grown ECTC on day 33 (**C**). Scale bar in A and C is 1 mm, and in B is 200 µ.

2.4. Data registration and processing

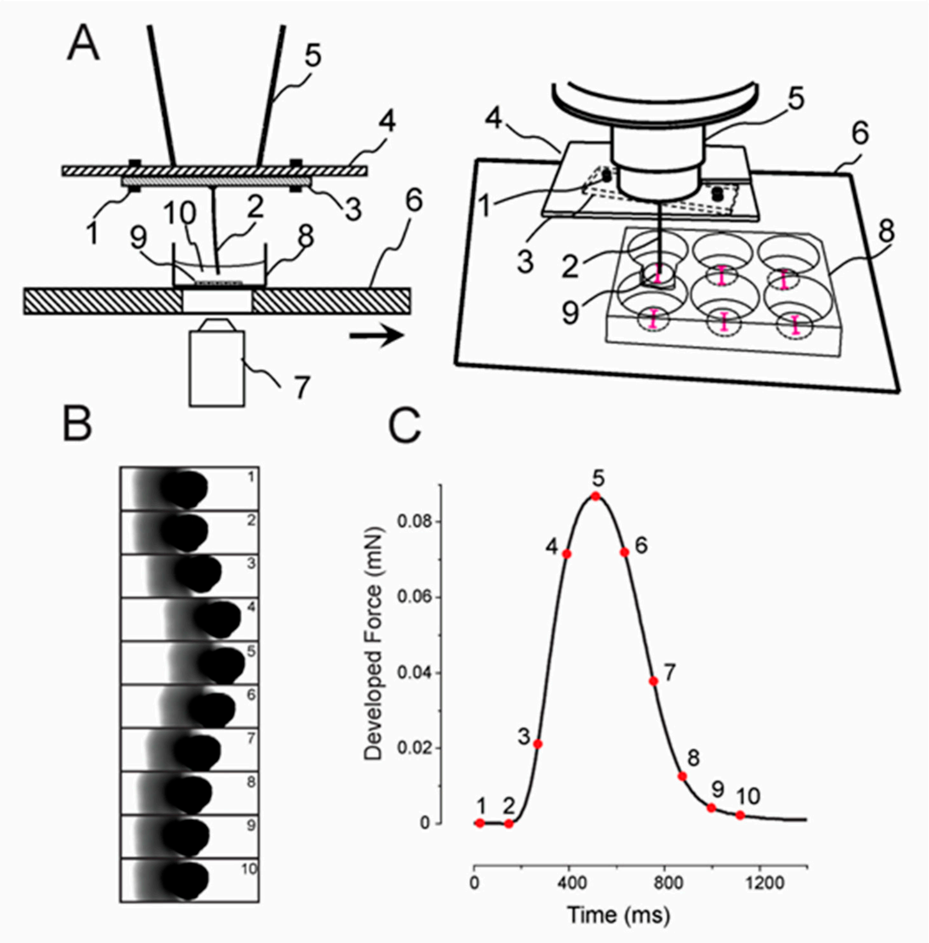

Figure S2. The I-Wire system for measuring the contractility of engineered cardiac tissue constructs (ECTCs). **A**) Diagram of the registration system: 1—magnet, 2—flexible probe, 3—cantilever, 4—cantilever support plate, 5—con-denser, 6—motorized stage, 7—lens, 8—well plate, and 9—insert with construct (red "I"). Adapted from Sulgin, et al., (2020) [33]. **B**) The sequence of images of the probe position during ECTC contraction; time scale is 121 ms/frame. **C**) Contraction time trace. Red dots correspond to frames in B. The sCMOS camera acquisition rate is 165 fps.

Figure S3 illustrates a force vector diagram affecting ECTC during relaxation and contraction (B). The developed force (Figures 3 and 4 in the body of the manuscript) was calculated as the difference of F_{t2} and F_{t1}. The force was determined based on flexible probe calibration[2].

[33] Sulgin, A.A.; Sidorova, T.N.; Sidorov, V.Y. Growth and characterization of a tissue-engineered construct from human coronary artery smooth muscle cells. *Biulleten Sib Meditsiny* **2020**, *19*, 85-95, doi:10.20538/1682-0363-2020-2-85-95.

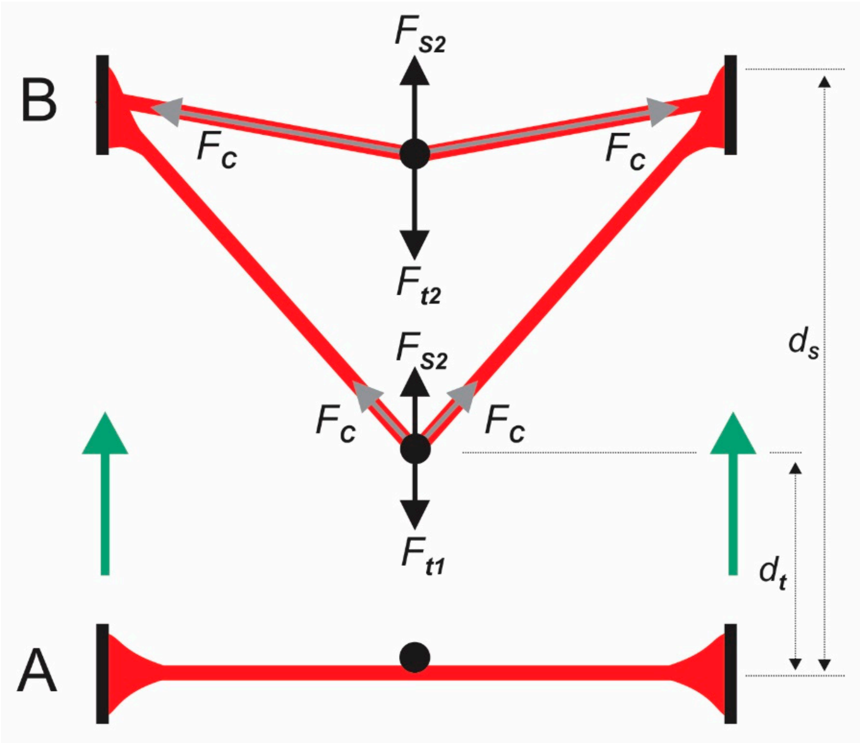

Figure S3. Schematic representation of force vectors exerted on and in the ECTC at the initial position A and after the shift to position B. There are two states at position B: relaxation when ECTC is extremely stretched and contraction when ECTC is shrunk. Black dot indicates the position of the flexible probe tip.

2.5. RNA preparation, next-generation sequencing (NGS), and gene set enrichment analysis (GSEA)

Three samples of each condition were utilized for analysis. The ECTC (1.06 ± 0.24 mg, N = 6) was detached from the device and transferred into a 1 mL vial containing 350 μL Lysis Buffer with 1% β-mercaptoethanol and homogenized with a plastic pestle of a cordless homogenizer (NC0493674, Fisher Scientific, USA). After centrifugation at 13,000 x g for 3 min, supernatant was removed, mixed thoroughly with the same volume of 70 % EtOH, applied to the RNeasy mini column and centrifuged at 10,000 x g for 1 min. Thereafter the mini column was washed once with 350 uL Wash Buffer I and two times with Wash Buffer II at 10,000 x g for 15 sec. To elute the RNA, nuclease-free water (up to 20 μL) was added directly onto the RNeasy silica gel membrane, and the column was centrifuged at 14,000xg for 1 min. Purified total RNA was stored at –80 °C. RNA samples were analyzed with the next-generation sequencing (NGS) method at multiplex paired-end 150 bp on the Illumina NovaSeq 6000 platform (NextSeq Instrument, Illumina, San Diego, CA, USA) (Vanderbilt Technologies for Advanced Genomics, Vanderbilt University Medical Center, Nashville, TN, USA). The intermediate analysis and read mapping to the Human Genome (hg38) were conducted with Illumina's Dragen RNA Seq pipeline.

We utilized the Gene Set Enrichment Analysis online tool (http://www.gsea-msigdb.org/) for the enrichment analysis of our differentially expressed gene groups. Gene names were copied into the Investigate Gene Set tool, where we selected the species (human) and specific ontology (biological process, cellular component, and molecular function) for the analysis. Significant terms associated with the up- or down-regulated gene set are presented along with enrichment score (– log10(q-value)) and numbers of genes. The genes with gene descriptions linked with certain terms are presented as overlap matrix maps, which together with tables of GO terms, gene counts, p-values, and FDRs are illustrated below in the next-generation sequencing section.

3.1. Growth and characterization of ECTCs

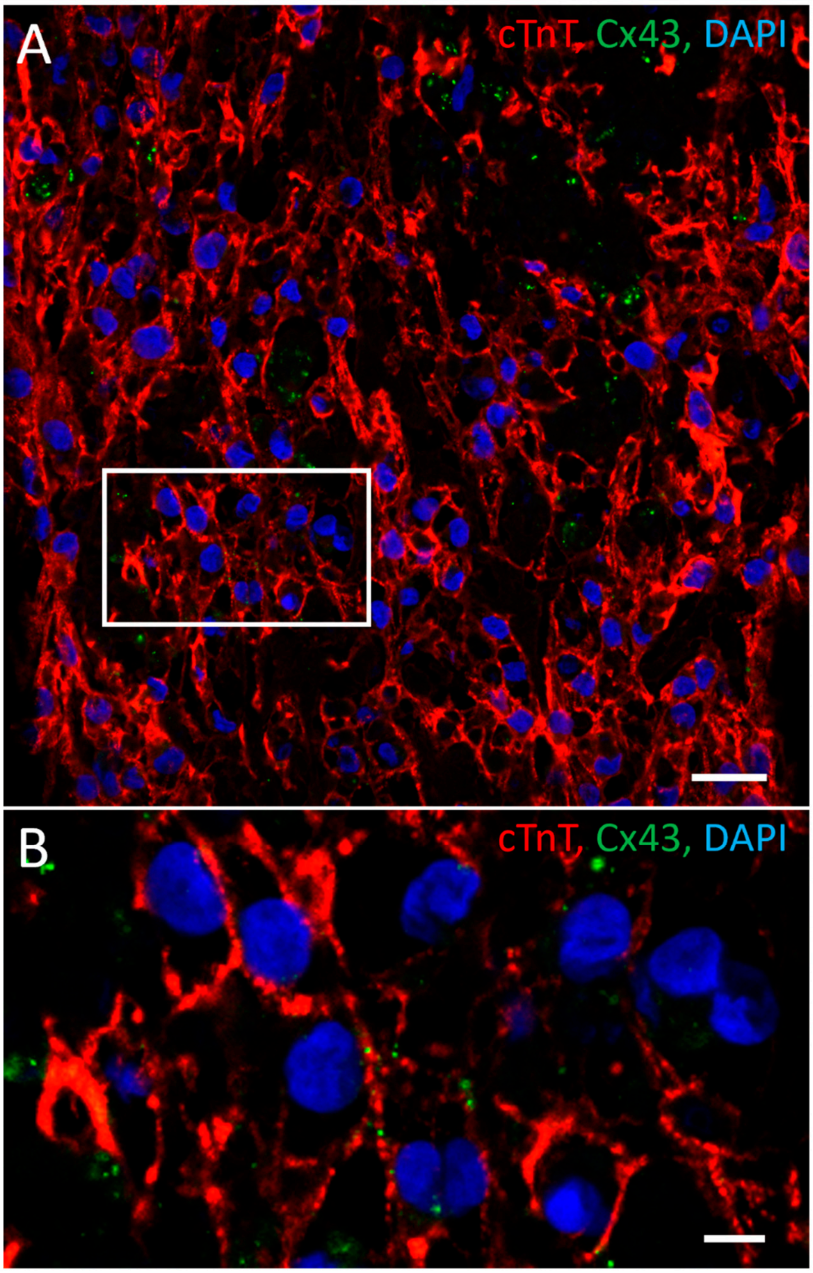

Figure S4. Histological imaging of an ECTC on Day 29. ECTC was stained against cardiac Troponin T (cTnT, red), connexin-43 (Cx43, green) and DAPI (blue). Scale bar is 20 mm in A and 5 mm in B.

3.2. Inotropic and chronotropic effects of rapamycin in CC3 and TSC constructs

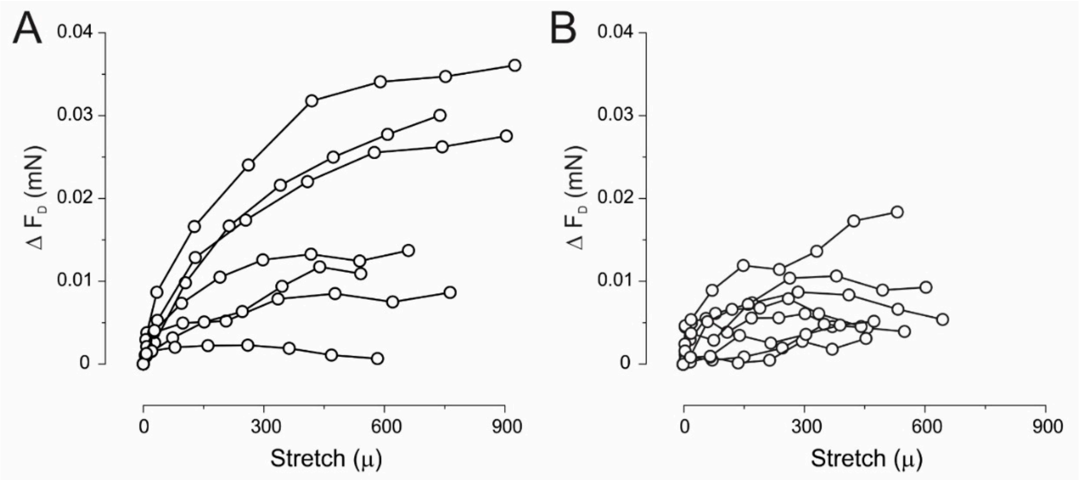

Figure S5. The change in the peak developed force (ΔF_D) in response to rapamycin as a function of the stretch of *N* = 7 CC3 ECTCs (**A**) and *N* = 8 TSP8-15 ECTCs (**B**). Each line represents a different construct that was individually differentiated.

3.3. Elastic properties

Young's modulus was calculated according to a modified strength/stress equation [31]

$$E = \frac{L_o * F_t * 2(\sqrt{(d_s - d_t)^2 + a^2})}{\pi D^2 * \Delta L * (d_s - d_t)},$$

where F_t is the force applied by the sensor, d_s is the distance between the original (A) and present moving platform position (B), d_t is the distance between the original and present position of the flexible probe tip, a is one-half of the length of the ECTC in a relaxed state, and D is the diameter of the ECTC. D was determined as a mean value of three measurements: at the middle point of the ECTC and at two points 1 mm away from the middle point on the left and right sides.

Figure S5 demonstrates the effect of rapamycin as a function of stretch in the CC3 and TSP8-15 ECTCs, while Figure S6 shows the analysis of ECTC elasticity in response to rapamycin.

[31] Sidorov, V.Y.; Samson, P.C.; Sidorova, T.N.; Davidson, J.M.; Lim, C.C.; Wikswo, J.P. I-Wire Heart-on-a-Chip I: Three-dimensional cardiac tissue constructs for physiology and pharmacology. *Acta Biomater* **2017**, *48*, 68-78, doi:10.1016/j.actbio.2016.11.009.

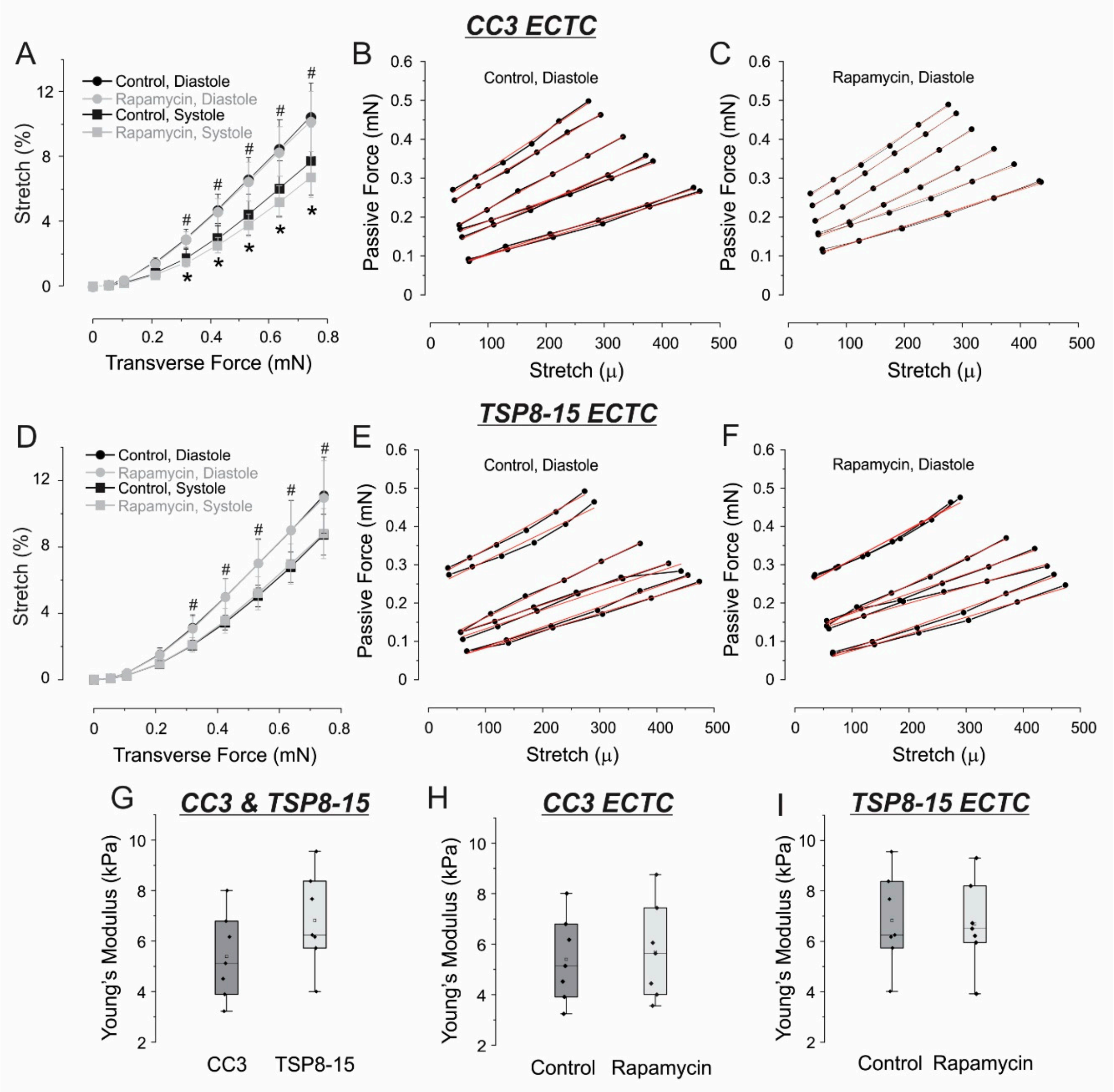

Figure S6. Effect of rapamycin on elastic properties of CC3 and TSP8-15 ECTCs. The average tension dependence of stretch during contraction, relaxation, and under 10 nM rapamycin is shown in (**A**) for CC3 ECTCs, and in (**B**) for TSP8-15 ECTCs. (**B, C, E, F**) Stretch–stress curves (black) and linear fits (red) used to calculate Young's modulus shown in (**G**), (**H**), (**I**). $^{*}P$ < 0.05 is for rapamycin relaxation compared with control relaxation, $^{\#}P$ < 0.001 is for control relaxation compared with control contraction. For all data, N = 7 ECTCs.

3.4. Next-generation sequencing (NGS) and gene ontology enrichment analysis of differentially expressed genes (DEGs)

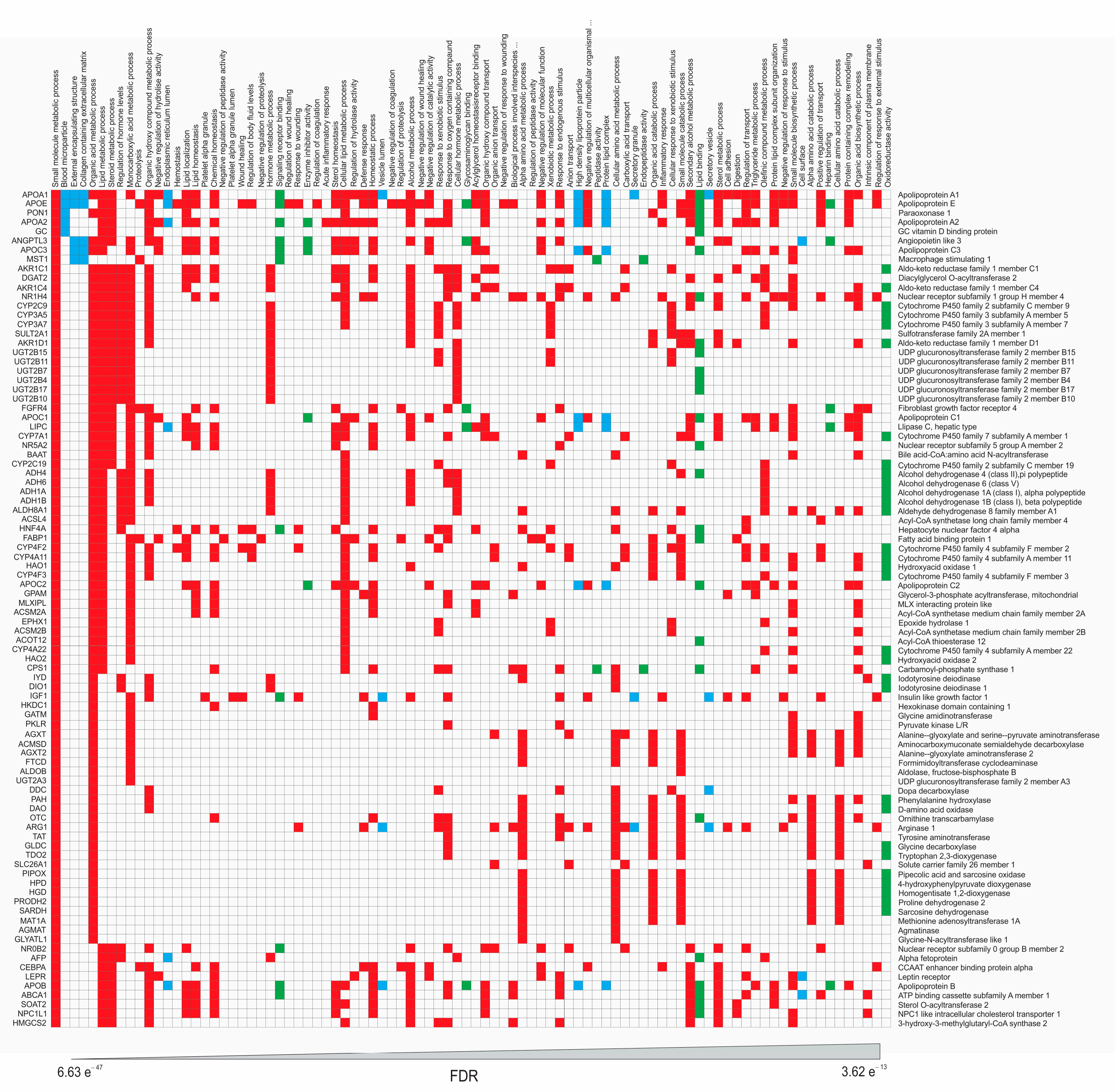

Figure S7. Comparison of CC3 and TSP8-15 ECTCs. The overlap matrix shows the relation between the top-scored GO terms and the corresponding up-regulated DEGs and their products.

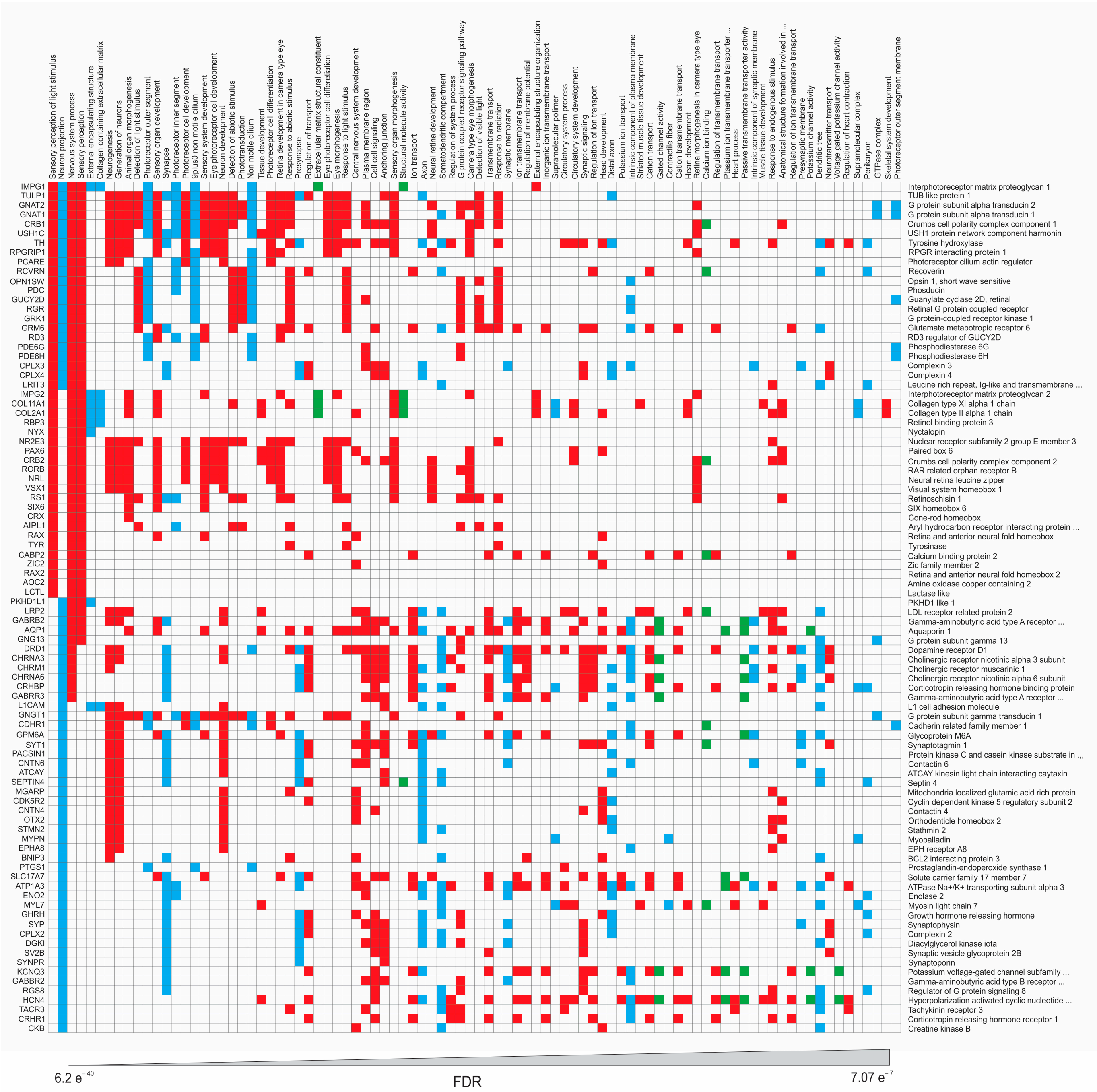

Figure S8. Comparison of CC3 and TSP8-15 ECTCs. The overlap matrix shows the relation between the top-scored GO terms and the corresponding down-regulated DEGs and their products.

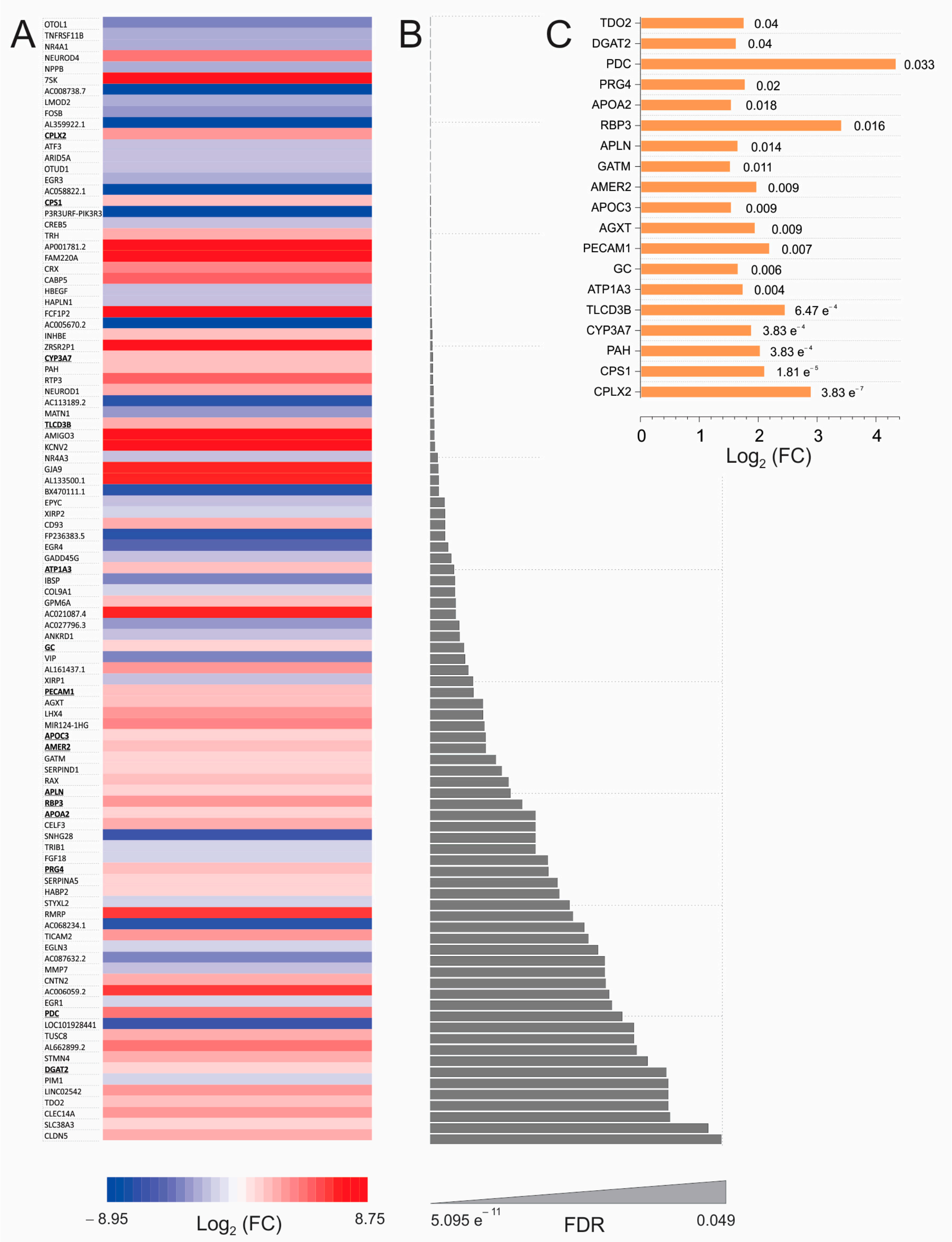

Figure S9. Gene expression in CC3 ECTCs in response to rapamycin. **A**) Fold change (FC) heat map. **B**) False discovery rate (FDR). **C**) Fold change of selected genes associated with the metabolic activity of fatty and amino acids. The numbers in C indicate FDR for each gene.

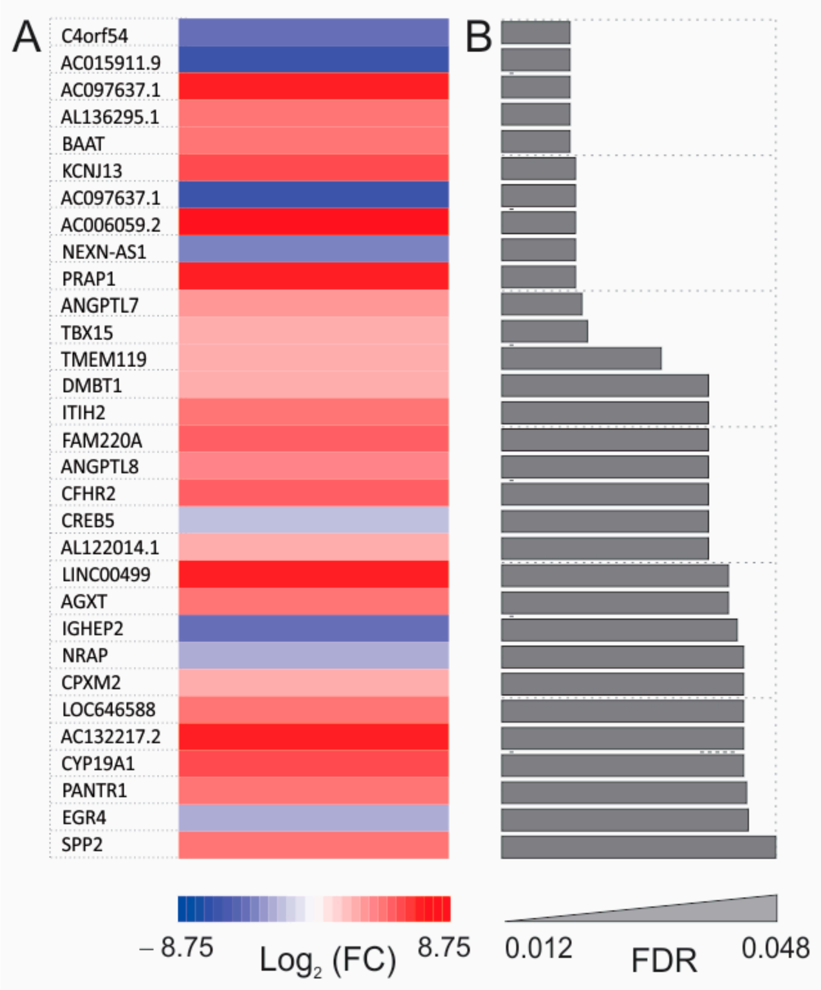

Figure S10. Gene expression in TSC8-15 ECTCs in response to rapamycin.

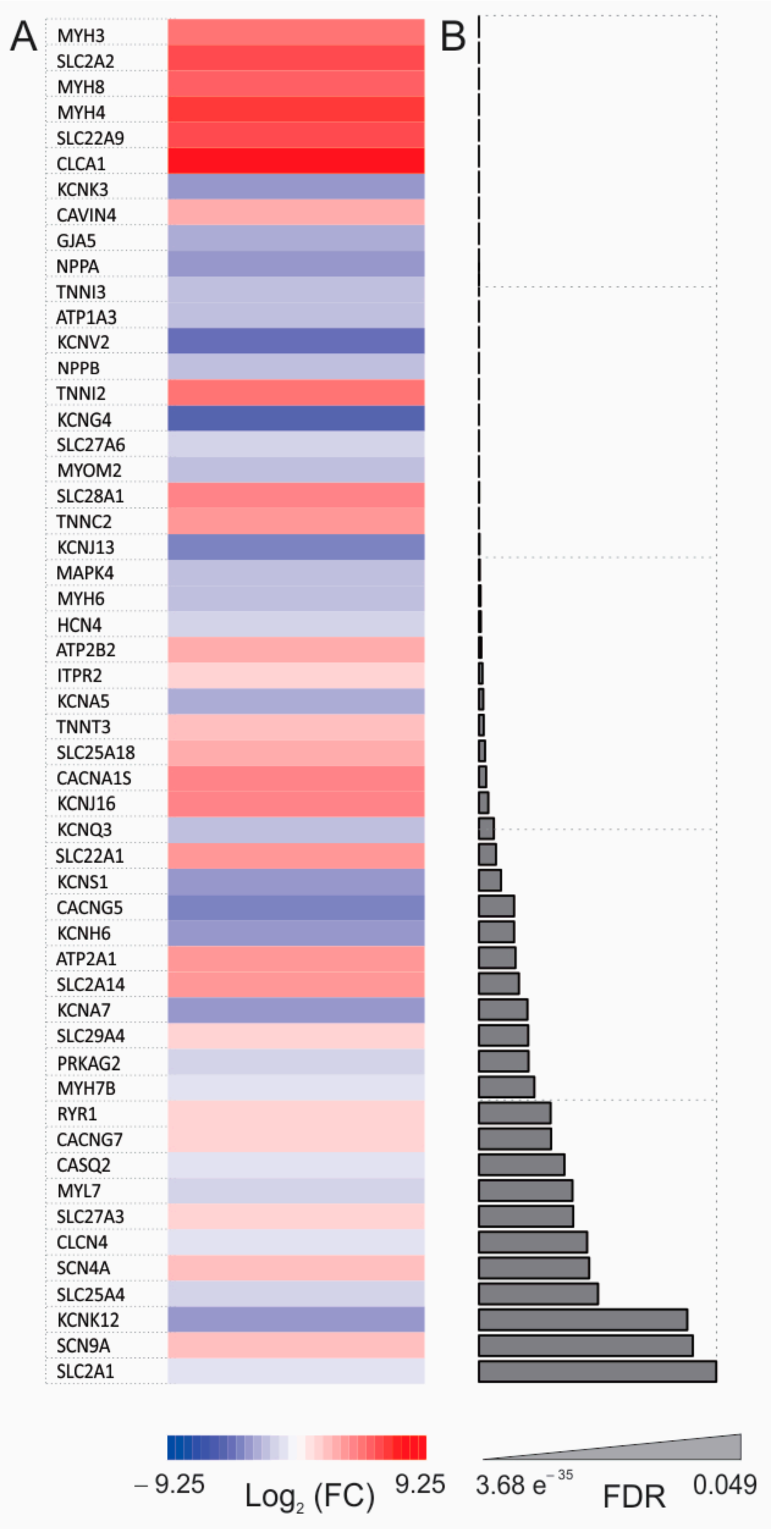

Figure S11. Cardiac-specific TSC8-15 ECTC genes differentially expressed relative to those in CC3.

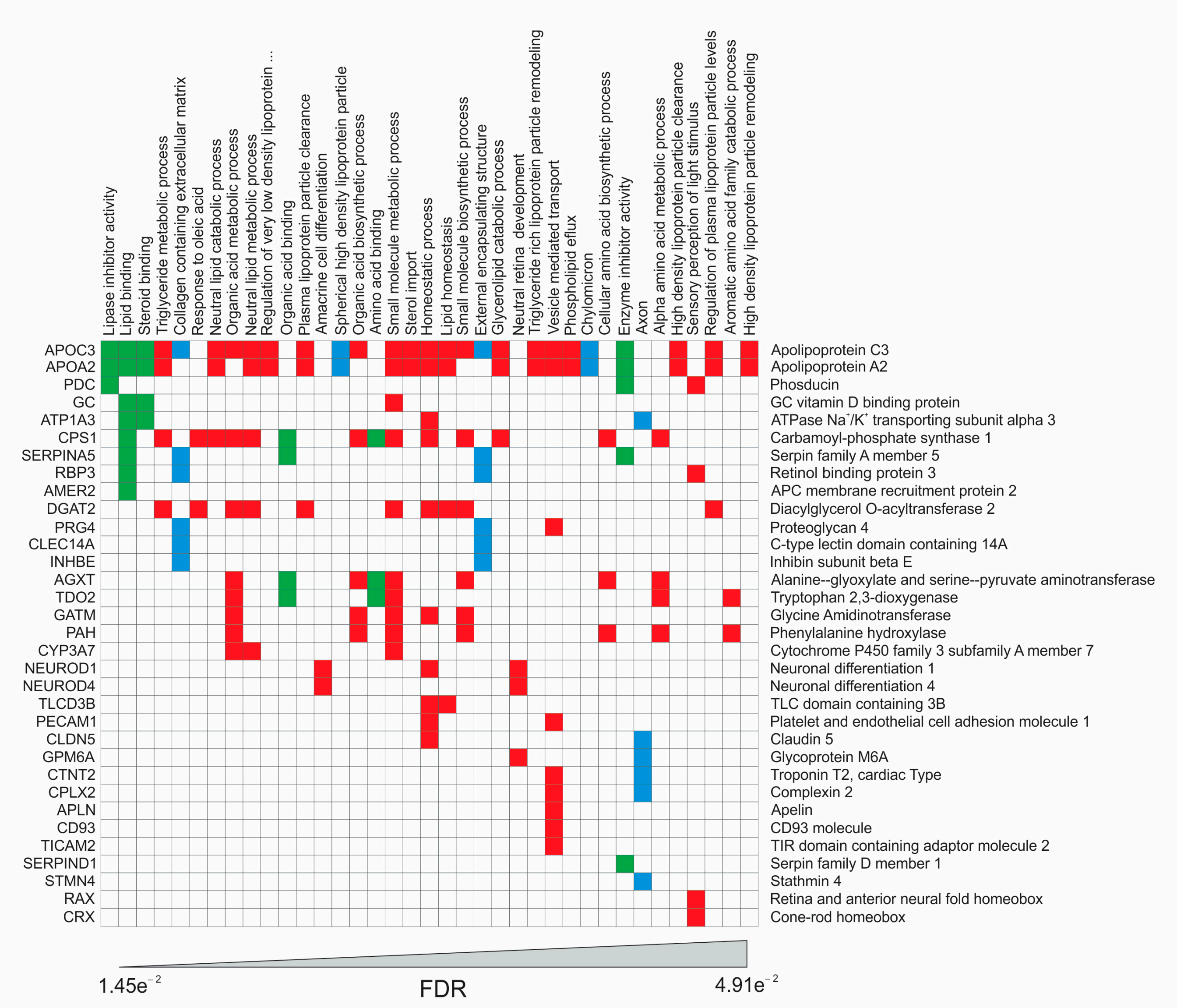

Figure S12. Comparison of CC3 and CC3/+rapamycin ECTCs. Overlap matrix of relationship between the top-scored GO terms and the corresponding up-regulated DEGs and their products.

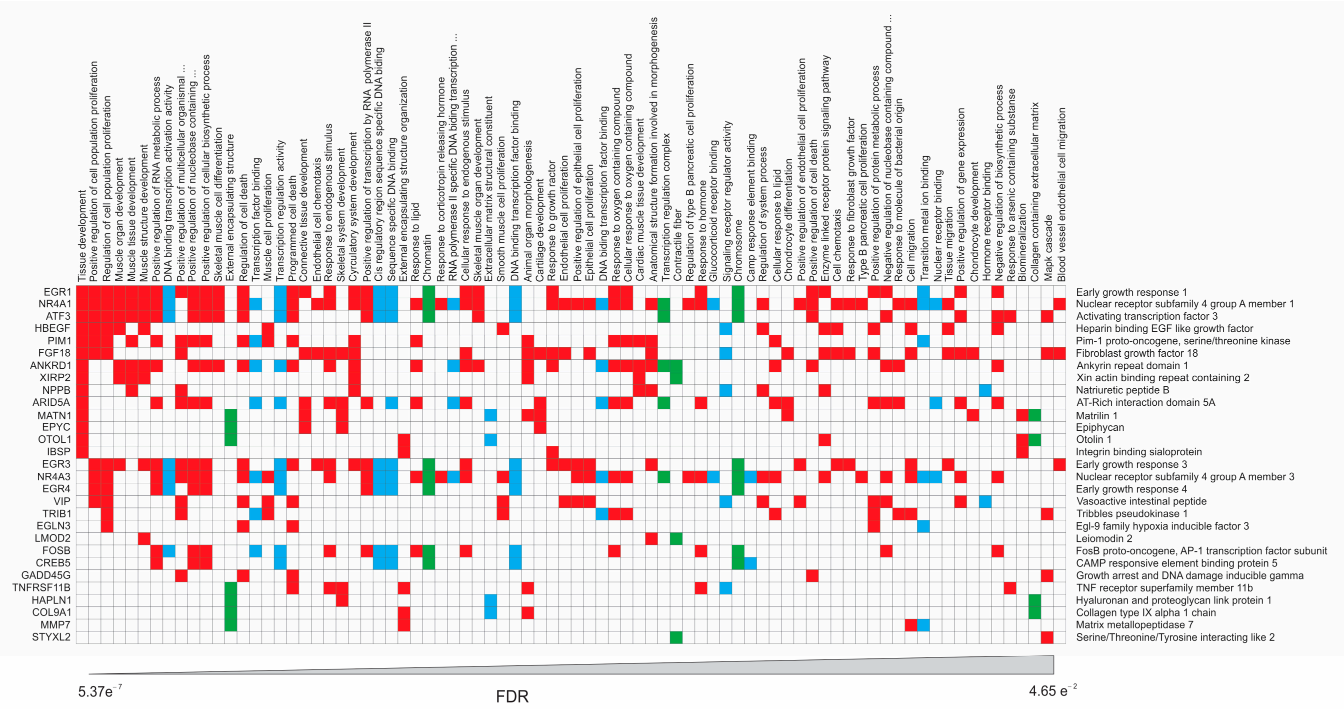

Figure S13. Comparison of CC3 and CC3/+rapamycin ECTCs. Overlap matrix of relationship between the top-scored GO terms and the corresponding down-regulated DEGs and their products.

Table S1. Up-regulated top-ranked 100 DEGs obtained by comparison of CC3 and TSP8-15 ECTCs.

Ensembl ID	NCBI Gene Symbol	Log (FC) *	Log (CPM) **	P-value	FDR***
ENSG00000151655.19	ITIH2	7.080177718	7.066942475	7.52E-76	4.56E-71
ENSG00000145321.13	GC	6.812928237	7.349195218	6.32E-70	1.91E-65
ENSG00000148702.15	HABP2	6.372574574	6.068026372	2.64E-67	5.34E-63
ENSG00000160870.14	CYP3A7	6.427414038	7.157346736	5.11E-66	7.75E-62
ENSG00000188488.14	SERPINA5	6.246218802	6.170445662	7.66E-66	9.28E-62
ENSG00000099937.11	SERPIND1	6.179735264	5.886667873	1.18E-63	1.19E-59
ENSG00000113889.14	KNG1	5.947514328	5.858651329	3.58E-60	2.91E-56
ENSG00000084674.14	APOB	6.668420108	8.135054312	3.84E-60	2.91E-56
ENSG00000171759.10	PAH	6.764222889	4.945064351	8.11E-60	5.46E-56
ENSG00000132855.5	ANGPTL3	6.833102296	4.894314958	2.10E-59	1.27E-55
ENSG00000169903.7	TM4SF4	6.121781643	5.459793378	3.49E-59	1.93E-55
ENSG00000110169.11	HPX	5.73386121	6.608638452	2.45E-58	1.24E-54
ENSG00000187758.8	ADH1A	8.395596196	4.259017959	2.70E-58	1.26E-54
ENSG00000180210.14	F2	6.080131422	5.358334257	1.69E-57	7.31E-54
ENSG00000081051.8	AFP	7.388830201	9.230283462	1.04E-56	4.20E-53
ENSG00000158874.11	APOA2	5.846165255	7.367801948	2.80E-56	1.06E-52
ENSG00000161944.16	ASGR2	6.550895341	4.883059312	4.11E-56	1.47E-52
ENSG00000055957.11	ITIH1	5.604481229	6.035290649	5.02E-56	1.69E-52
ENSG00000167711.13	SERPINF2	5.53014612	6.317384883	1.44E-55	4.61E-52
ENSG00000055955.17	ITIH4	6.067962717	5.211050169	2.13E-55	6.44E-52
ENSG00000122194.18	PLG	5.422671535	6.123843228	1.09E-53	3.01E-50
ENSG00000091583.11	APOH	5.419095364	6.86753896	5.41E-53	1.43E-49
ENSG00000138207.14	RBP4	5.221801547	6.54539359	5.35E-51	1.35E-47
ENSG00000139269.3	INHBE	5.436375101	5.489677967	1.57E-50	3.81E-47
ENSG00000106927.12	AMBP	5.921542526	8.184416065	2.08E-50	4.84E-47
ENSG00000109072.14	VTN	5.867224372	8.268621643	5.27E-49	1.18E-45
ENSG00000187045.18	TMPRSS6	5.257182984	5.382324361	4.79E-47	1.04E-43
ENSG00000145192.13	AHSG	6.173604836	8.957492912	1.79E-46	3.74E-43
ENSG00000163586.10	FABP1	5.379900076	5.138270318	3.80E-46	7.69E-43
ENSG00000171557.17	FGG	5.691278204	8.40602984	1.01E-45	1.97E-42
ENSG00000171560.16	FGA	6.178909736	9.070694574	1.40E-45	2.66E-42
ENSG00000162267.12	ITIH3	5.16357672	5.333566943	3.67E-45	6.74E-42
ENSG00000136872.20	ALDOB	4.823432743	5.978629327	1.05E-44	1.88E-41
ENSG00000100665.12	SERPINA4	6.622885137	4.234798567	1.73E-44	2.99E-41
ENSG00000196620.10	UGT2B15	8.818863645	3.628898466	3.66E-44	6.17E-41
ENSG00000130829.18	DUSP9	5.291161814	5.026442969	1.01E-43	1.65E-40
ENSG00000170099.6	SERPINA6	5.849637334	4.569253836	2.10E-43	3.35E-40
ENSG00000130208.9	APOC1	5.002962234	5.318057643	3.23E-43	5.02E-40
ENSG00000171564.11	FGB	5.767963194	8.830241677	3.91E-43	5.93E-40
ENSG00000127831.11	VIL1	6.629900679	4.140847675	9.25E-43	1.37E-39
ENSG00000198734.11	F5	6.351291322	4.24908232	1.49E-42	2.14E-39
ENSG00000118271.10	TTR	4.702522443	6.987864881	2.33E-42	3.29E-39
ENSG00000156096.14	UGT2B4	5.055254953	5.11402368	6.93E-42	9.33E-39
ENSG00000257017.9	HP	4.599874148	6.641308909	7.12E-42	9.38E-39
ENSG00000105707.14	HPN	5.514694162	4.691003631	7.50E-42	9.68E-39
ENSG00000110245.12	APOC3	4.566072067	6.233787548	1.24E-41	1.53E-38
ENSG00000213886.4	UBD	4.854560359	5.357854062	2.21E-41	2.68E-38
ENSG00000117601.13	SERPINC1	5.31706638	4.583343898	3.05E-38	3.63E-35

ENSG00000109063.15	MYH3	4.806562416	5.0663884	3.16E-38	3.68E-35
ENSG00000151790.9	TDO2	5.786059897	4.253615555	8.41E-38	9.62E-35
ENSG00000148584.15	A1CF	6.395533983	3.970996222	1.11E-37	1.24E-34
ENSG00000197249.13	SERPINA1	5.942931667	9.919874947	3.14E-37	3.46E-34
ENSG00000136881.11	BAAT	6.182584755	3.978110343	2.79E-36	3.02E-33
ENSG00000214548.18	MEG3	12.83724408	3.01004581	4.32E-36	4.59E-33
ENSG00000229314.5	ORM1	4.221394245	5.829289536	5.91E-36	6.17E-33
ENSG00000123838.11	C4BPA	6.680343946	3.769103463	6.87E-36	7.06E-33
ENSG00000172482.5	AGXT	5.450587661	4.30375696	1.33E-35	1.34E-32
ENSG00000118137.9	APOA1	5.09121088	8.912665551	2.43E-35	2.42E-32
ENSG00000109181.12	UGT2B10	6.673160731	3.726974945	4.40E-35	4.30E-32
ENSG00000228278.4	ORM2	5.505151129	4.17196366	5.20E-34	5.00E-31
ENSG00000184697.7	CLDN6	6.164836011	3.822097549	2.50E-33	2.33E-30
ENSG00000163581.14	SLC2A2	6.05429229	3.843304567	4.55E-33	4.17E-30
ENSG00000021826.16	CPS1	4.00210961	5.823075745	5.04E-33	4.56E-30
ENSG00000169562.12	GJB1	5.318279063	4.195024633	9.54E-33	8.50E-30
ENSG00000118520.15	ARG1	5.646291062	3.986610321	2.30E-32	1.99E-29
ENSG00000196136.17	SERPINA3	4.141043779	7.642549175	5.36E-32	4.58E-29
ENSG00000166035.11	LIPC	5.621260187	3.983757907	6.40E-32	5.39E-29
ENSG00000123561.15	SERPINA7	5.396608304	4.071371189	1.26E-31	1.04E-28
ENSG00000104760.17	FGL1	5.739263969	3.811362856	4.39E-30	3.60E-27
ENSG00000134240.11	HMGCS2	4.452265211	4.64063735	5.15E-30	4.17E-27
ENSG00000133020.4	MYH8	5.058193388	4.181694182	7.13E-30	5.69E-27
ENSG00000101076.16	HNF4A	5.699196102	3.800654495	7.22E-30	5.69E-27
ENSG00000137204.14	SLC22A7	4.647230038	4.428161898	1.21E-29	9.41E-27
ENSG00000168530.16	MYL1	5.452525972	3.914498887	1.83E-29	1.41E-26
ENSG00000131187.9	F12	4.808990618	4.224653565	1.24E-28	9.40E-26
ENSG00000166278.15	C2	3.973892164	5.03387833	2.49E-28	1.86E-25
ENSG00000021852.13	C8B	5.627095777	3.69255699	8.20E-28	6.06E-25
ENSG00000141505.12	ASGR1	4.322852188	4.535663134	1.14E-27	8.36E-25
ENSG00000160862.13	AZGP1	4.163954446	4.685750347	1.71E-27	1.24E-24
ENSG00000139540.12	SLC39A5	5.470784648	3.745018628	1.79E-27	1.27E-24
ENSG00000080618.16	CPB2	5.939832605	3.496351711	7.53E-27	5.31E-24
ENSG00000171766.16	GATM	3.460426814	6.263267501	1.13E-26	7.91E-24
ENSG00000264424.1	MYH4	6.518011252	3.253815062	1.50E-26	1.03E-23
ENSG00000132703.4	APCS	5.207301195	3.803790357	1.93E-26	1.31E-23
ENSG00000163631.17	ALB	6.105851017	12.55480804	3.79E-26	2.55E-23
ENSG00000224389.9	C4B	3.674921412	7.782758612	7.95E-26	5.30E-23
ENSG00000129988.6	LBP	5.155829123	3.778002158	1.21E-25	7.98E-23
ENSG00000137491.15	SLCO2B1	5.535053019	3.560464369	2.13E-25	1.39E-22
ENSG00000268230.5	AC012313.3	5.536841158	3.551443139	4.22E-25	2.72E-22
ENSG00000149742.10	SLC22A9	5.951998484	3.34237026	8.07E-25	5.09E-22
ENSG00000160282.14	FTCD	5.014014258	3.751741776	1.93E-24	1.21E-21
ENSG00000135220.11	UGT2A3	6.651731696	3.082492869	2.03E-24	1.26E-21
ENSG00000100604.13	CHGA	3.344415967	5.614269343	4.97E-24	3.01E-21
ENSG00000244414.6	CFHR1	5.025965176	3.7144786	5.11E-24	3.06E-21
ENSG00000053438.11	NNAT	-5.906734987	3.303596229	5.96E-24	3.54E-21
ENSG00000188338.15	SLC38A3	-3.700126104	4.824105495	7.08E-24	4.17E-21
ENSG00000122787.15	AKR1D1	-6.777664832	2.983229862	9.80E-24	5.71E-21
ENSG00000174827.13	PDZK1	-5.756212686	3.336004772	1.27E-23	7.33E-21
ENSG00000076770.14	MBNL3	-3.241439801	5.884547581	1.75E-23	1.00E-20
ENSG00000234906.10	APOC2	-5.593819685	3.39352338	1.80E-23	1.02E-20

* Fold change; ** counts per million; ***false discovery rate.

Table S2. Down-regulated top-ranked 100 DEGs obtained by comparison of CC3 and TSP8-15 ECTCs.

Ensembl ID	NCBI Gene Symbol	Log (FC) *	Log (CPM) **	P-value	FDR***
ENSG00000179914.5	ITLN1	7.107865393	6.610336369	5.46E-55	1.58E-51
ENSG00000109047.8	RCVRN	9.44093939	4.367962851	2.62E-42	3.60E-39
ENSG00000108018.15	SORCS1	11.02110558	4.080372146	1.09E-41	1.37E-38
ENSG00000129221.16	AIPL1	6.874046088	4.35656378	1.08E-33	1.02E-30
ENSG00000182447.4	OTOL1	6.617152726	4.270806966	1.52E-32	1.34E-29
ENSG00000124159.15	MATN4	3.937740754	6.94090654	5.96E-25	3.80E-22
ENSG00000265203.2	RBP3	5.694052558	4.063698107	3.04E-24	1.86E-21
ENSG00000156395.13	SORCS3	11.79774999	3.00649355	2.11E-22	1.12E-19
ENSG00000160678.11	S100A1	5.715307461	3.573530398	1.12E-19	5.49E-17
ENSG00000129535.12	NRL	3.883326949	4.705105166	3.96E-19	1.91E-16
ENSG00000169085.13	C8orf46	4.828155846	3.936909318	7.40E-19	3.44E-16
ENSG00000145681.11	HAPLN1	3.085460381	6.409227656	9.01E-18	3.87E-15
ENSG00000081148.12	IMPG2	3.719511969	4.603565622	2.19E-17	9.22E-15
ENSG00000171303.7	KCNK3	3.607010576	4.688355553	3.65E-17	1.49E-14
ENSG00000102104.8	RS1	9.170361078	2.672166136	6.04E-17	2.40E-14
ENSG00000164764.11	SBSPON	5.439159968	3.395757616	4.18E-16	1.58E-13
ENSG00000132031.13	MATN3	4.038967386	4.031784065	9.93E-16	3.65E-13
ENSG00000148600.15	CDHR1	3.669668401	4.37292458	1.09E-15	3.94E-13
ENSG00000116703.14	PDC	5.325833327	3.277254978	4.84E-15	1.71E-12
ENSG00000165566.12	AMER2	3.339993893	4.614730613	5.24E-15	1.83E-12
ENSG00000255823.5	MTRNR2L8	5.618485952	3.202362432	5.47E-15	1.90E-12
ENSG00000123307.4	NEUROD4	3.464340888	4.4524892	6.39E-15	2.21E-12
ENSG00000105392.16	CRX	4.091721525	3.895676499	7.27E-15	2.46E-12
ENSG00000111664.10	GNB3	3.247585157	4.685020076	9.24E-15	3.06E-12
ENSG00000104888.10	SLC17A7	4.234550813	3.70998933	2.89E-14	9.16E-12
ENSG00000105507.3	CABP5	4.342398426	3.620754164	5.15E-14	1.58E-11
ENSG00000162992.3	NEUROD1	3.293235246	4.428568883	8.87E-14	2.65E-11
ENSG00000184845.4	DRD1	4.630875148	3.381756223	1.45E-13	4.24E-11
ENSG00000157766.17	ACAN	2.81304901	7.831588017	1.54E-13	4.47E-11
ENSG00000205038.12	PKHD1L1	4.596770033	3.364353353	3.64E-13	1.02E-10
ENSG00000102003.10	SYP	2.811788026	4.98457268	5.10E-13	1.41E-10
ENSG00000144619.15	CNTN4	7.191451816	2.42126451	5.22E-13	1.44E-10
ENSG00000105251.10	SHD	2.888298678	4.824148271	5.84E-13	1.59E-10
ENSG00000167768.4	KRT1	7.924116778	2.112652949	9.28E-13	2.44E-10
ENSG00000136110.13	CNMD	3.122012638	4.397131878	1.02E-12	2.65E-10
ENSG00000265107.3	GJA5	2.569044958	5.435727858	1.69E-12	4.26E-10
ENSG00000077009.13	NMRK2	3.587441528	3.903852241	1.81E-12	4.53E-10
ENSG00000080166.16	DCT	3.030169894	4.449213332	2.29E-12	5.70E-10
ENSG00000186832.9	KRT16	2.491563934	5.594109986	3.09E-12	7.51E-10
ENSG00000175206.10	NPPA	3.289084319	9.862623879	3.20E-12	7.73E-10
ENSG00000161281.11	COX7A1	4.373967184	3.313019782	3.59E-12	8.63E-10
ENSG00000198812.5	LRRC10	2.396243364	6.122086338	4.95E-12	1.18E-09
ENSG00000112041.13	TULP1	4.553174732	3.167966745	8.71E-12	2.00E-09
ENSG00000144331.20	ZNF385B	2.789855939	4.674412716	9.32E-12	2.13E-09
ENSG00000165588.17	OTX2	2.712506563	4.78247572	1.15E-11	2.63E-09
ENSG00000150625.16	GPM6A	2.513250343	5.233908133	1.18E-11	2.68E-09
ENSG00000145824.12	CXCL14	3.014593286	4.248908608	2.07E-11	4.57E-09
ENSG00000129991.13	TNNI3	2.429134045	7.462872921	2.12E-11	4.68E-09

ENSG00000081842.18	PCDHA6	3.787529265	3.537914075	2.56E-11	5.55E-09
ENSG00000128918.15	ALDH1A2	3.153369381	4.100680819	2.93E-11	6.28E-09
ENSG00000180660.8	MAB21L1	2.779192962	4.546027304	2.95E-11	6.29E-09
ENSG00000145920.15	CPLX2	2.617332366	4.779138045	4.63E-11	9.51E-09
ENSG00000162706.13	CADM3	2.531279156	4.951936057	4.99E-11	1.01E-08
ENSG00000173976.15	RAX2	5.337375856	2.700222871	5.75E-11	1.15E-08
ENSG00000105409.19	ATP1A3	2.338203765	5.483545515	6.60E-11	1.30E-08
ENSG00000139053.3	PDE6H	10.40860678	1.523185985	7.14E-11	1.39E-08
ENSG00000166523.8	CLEC4E	8.199664836	-1.103692402	7.29E-11	1.41E-08
ENSG00000142538.2	PTH2	8.868966051	-0.589804727	1.12E-10	2.11E-08
ENSG00000092758.18	COL9A3	2.627255634	8.688760041	1.26E-10	2.37E-08
ENSG00000213578.6	CPLX3	5.344791508	2.607661101	1.46E-10	2.71E-08
ENSG00000134438.10	RAX	2.490794507	4.865377726	1.49E-10	2.75E-08
ENSG00000163092.20	XIRP2	2.217414016	6.74044674	1.60E-10	2.92E-08
ENSG00000163833.8	FBXO40	2.260646544	5.572544969	1.67E-10	3.03E-08
ENSG00000168263.9	KCNV2	4.577793475	2.936003721	1.80E-10	3.27E-08
ENSG00000186847.6	KRT14	2.263215425	5.401176377	2.82E-10	5.01E-08
ENSG00000149926.13	FAM57B	2.787052953	4.273118809	3.24E-10	5.66E-08
ENSG00000104879.5	CKM	2.317699472	7.759191511	3.35E-10	5.81E-08
ENSG00000121454.6	LHX4	3.641094567	3.418703532	4.40E-10	7.50E-08
ENSG00000127588.5	GNG13	7.800666141	-1.233640972	5.83E-10	9.69E-08
ENSG00000262633.2	AC005670.2	9.011688226	-0.334458238	6.71E-10	1.11E-07
ENSG00000102313.9	ITIH6	2.974063204	3.943662681	7.82E-10	1.29E-07
ENSG00000164236.12	ANKRD33B	2.310711016	5.024229382	8.85E-10	1.45E-07
ENSG00000236782.7	AL391650.1	4.267622737	2.949175801	9.72E-10	1.58E-07
ENSG00000134594.5	RAB33A	4.075342195	3.062403757	1.00E-09	1.62E-07
ENSG00000160307.10	S100B	2.535923565	4.483962381	1.23E-09	1.96E-07
ENSG00000134115.13	CNTN6	8.616814545	-0.476273087	1.25E-09	1.99E-07
ENSG00000180332.6	KCTD4	9.920087806	0.337332401	1.32E-09	2.10E-07
ENSG00000279117.1	AP001972.5	7.708570445	-1.114520124	1.89E-09	2.90E-07
ENSG00000113262.16	GRM6	5.335594819	2.337237074	2.93E-09	4.35E-07
ENSG00000120937.9	NPPB	2.043106972	6.916631311	3.92E-09	5.68E-07
ENSG00000112280.16	COL9A1	2.247948534	8.198305613	4.06E-09	5.87E-07
ENSG00000183305.13	MAGEA2B	7.502370298	-1.654938346	4.17E-09	6.01E-07
ENSG00000081479.15	LRP2	3.12561995	3.623877993	4.36E-09	6.26E-07
ENSG00000095303.17	PTGS1	3.49396576	3.319237853	4.74E-09	6.79E-07
ENSG00000138347.15	MYPN	2.247042538	4.862743673	5.90E-09	8.28E-07
ENSG00000139767.10	SRRM4	3.595794564	3.197937636	7.29E-09	1.00E-06
ENSG00000165023.7	DIRAS2	2.901710719	3.777070604	8.41E-09	1.15E-06
ENSG00000152256.13	PDK1	1.957079611	6.681086899	8.49E-09	1.16E-06
ENSG00000160539.6	PLPP7	2.037607411	5.419037265	8.85E-09	1.20E-06
ENSG00000188086.15	PRSS45	7.329331428	-1.462014884	1.11E-08	1.48E-06
ENSG00000163536.12	SERPINI1	1.995233959	5.516782924	1.24E-08	1.65E-06
ENSG00000197106.7	SLC6A17	3.151387208	3.500494217	1.25E-08	1.65E-06
ENSG00000244094.2	SPRR2F	5.723956738	2.014316361	1.29E-08	1.69E-06
ENSG00000122176.12	FMOD	1.925861416	6.59272789	1.30E-08	1.71E-06
ENSG00000204866.8	IGFL2	5.948002021	-0.827274478	1.35E-08	1.76E-06
ENSG00000083782.8	EPYC	2.127905154	4.963602136	1.50E-08	1.96E-06
ENSG00000241158.7	ADAMTS9-AS1	4.959464148	-1.279736676	1.66E-08	2.12E-06
ENSG00000152137.6	HSPB8	1.915937914	5.900946041	2.04E-08	2.58E-06
ENSG00000204248.10	COL11A2	2.273800039	8.813491879	2.11E-08	2.66E-06
ENSG00000139219.19	COL2A1	2.786843996	10.82434421	2.19E-08	2.74E-06

* Fold change; ** counts per million; ***false discovery rate.

Table S3. DEGs detected in CC3 ECTCs in response to application of rapamycin.

Ensembl ID	NCBI Gene Symbol	Log (FC) *	Log (CPM) **	P-value	FDR***
ENSG00000182447.4	OTOL1	-4.154721301	4.270806966	1.25E-15	5.10E-11
ENSG00000164761.9	TNFRSF11B	-3.019479086	5.672134522	1.68E-15	5.10E-11
ENSG00000123358.20	NR4A1	-2.833973701	6.279796671	1.44E-14	2.92E-10
ENSG00000123307.4	NEUROD4	4.394671094	4.4524892	2.48E-14	3.75E-10
ENSG00000120937.9	NPPB	-2.660615501	6.916631311	1.00E-12	1.22E-08
ENSG00000202198.1	RF00100	8.720947604	-1.481347806	1.79E-12	1.81E-08
ENSG00000273420.1	AC008738.7	-8.901185912	-0.886103403	1.06E-11	9.19E-08
ENSG00000170807.12	LMOD2	-2.552458973	7.463603526	2.11E-11	1.60E-07
ENSG00000125740.14	FOSB	-3.527274802	3.805949506	3.28E-11	2.21E-07
ENSG00000264545.2	AL359922.1	-8.2054863	-1.380677486	6.35E-11	3.83E-07
ENSG00000145920.15	CPLX2	2.908028325	4.779138045	6.95E-11	3.83E-07
ENSG00000162772.17	ATF3	-2.263016871	6.846686491	4.89E-10	2.47E-06
ENSG00000196843.16	ARID5A	-2.208322403	6.336000482	8.10E-10	3.78E-06
ENSG00000165312.6	OTUD1	-2.211117416	5.990192673	9.55E-10	4.13E-06
ENSG00000179388.9	EGR3	-2.655608972	4.389311905	2.44E-09	9.84E-06
ENSG00000282278.1	AC058822.1	-8.076572635	-2.033604153	4.24E-09	1.60E-05
ENSG00000021826.16	CPS1	2.117772719	5.823075745	5.07E-09	1.81E-05
ENSG00000278139.1	AL358075.4	-8.072495187	-2.058615657	5.95E-09	2.00E-05
ENSG00000146592.17	CREB5	-2.209810312	5.126674918	1.14E-08	3.65E-05
ENSG00000170893.4	TRH	2.402126326	4.738049417	1.74E-08	5.27E-05
ENSG00000258529.5	AP001781.2	8.004349268	-0.673563019	2.19E-08	6.13E-05
ENSG00000178397.13	FAM220A	7.277779018	1.408477151	2.23E-08	6.13E-05
ENSG00000105392.16	CRX	3.718283675	3.895676499	2.65E-08	6.79E-05
ENSG00000105507.3	CABP5	4.782436246	3.620754164	2.69E-08	6.79E-05
ENSG00000113070.8	HBEGF	-1.981641916	6.107902705	2.99E-08	7.24E-05
ENSG00000145681.11	HAPLN1	-1.962109338	6.409227656	4.12E-08	9.59E-05
ENSG00000228638.1	FCF1P2	7.452325897	-1.037840828	6.34E-08	0.000142296
ENSG00000262633.2	AC005670.2	-8.036361642	-0.334458238	1.07E-07	0.000232226
ENSG00000139269.3	INHBE	1.939362475	5.489677967	1.37E-07	0.000285803
ENSG00000212643.3	ZRSR2P1	7.367665609	-0.951014262	1.45E-07	0.000292812
ENSG00000160870.14	CYP3A7	1.894447865	7.157346736	2.00E-07	0.000383332
ENSG00000171759.10	PAH	2.0426916	4.945064351	2.05E-07	0.000383332
ENSG00000163825.4	RTP3	4.924410764	-1.881047592	2.09E-07	0.000383332
ENSG00000162992.3	NEUROD1	2.470431998	4.428568883	2.85E-07	0.000507627
ENSG00000262880.1	AC113189.2	-6.98285043	-1.63007436	3.10E-07	0.000525201
ENSG00000162510.6	MATN1	-3.251221516	3.152057932	3.12E-07	0.000525201
ENSG00000149926.13	FAM57B	2.465960097	4.273118809	3.95E-07	0.000646912
ENSG00000176020.9	AMIGO3	7.22166725	-1.95412689	4.06E-07	0.000646912
ENSG00000168263.9	KCNV2	7.164305016	2.936003721	4.62E-07	0.000718075
ENSG00000119508.18	NR4A3	-2.396919359	3.976689575	7.90E-07	0.001197402
ENSG00000131233.10	GJA9	6.858525017	-1.551583905	9.06E-07	0.001338859
ENSG00000285547.1	-	6.849982934	-1.413841812	9.60E-07	0.001384681
ENSG00000288258.1	-	-6.797603396	-1.671717379	9.88E-07	0.001393078
ENSG00000083782.8	EPYC	-1.874035805	4.963602136	1.74E-06	0.0023942
ENSG00000163092.20	XIRP2	-1.706940972	6.74044674	1.89E-06	0.002464848
ENSG00000125810.10	-	2.332755192	3.917893444	1.91E-06	0.002464848
ENSG00000281181.1	FP236383.3	-6.69953791	-1.217803187	1.91E-06	0.002464848
ENSG00000135625.8	EGR4	-5.252200775	0.095984035	2.38E-06	0.003002239
ENSG00000130222.11	GADD45G	-2.063775248	4.351820471	2.85E-06	0.003527971

ENSG00000105409.19	ATP1A3	1.750786367	5.483545515	3.29E-06	0.00398595
ENSG00000029559.7	IBSP	-3.911307866	-1.788131401	3.53E-06	0.004147019
ENSG00000112280.16	COL9A1	-1.832069963	8.198305613	3.56E-06	0.004147019
ENSG00000150625.16	GPM6A	1.794735993	5.233908133	3.79E-06	0.004295471
ENSG00000261434.1	AC021087.4	6.708635085	-1.044571138	3.83E-06	0.004295471
ENSG00000262304.2	AC027796.3	-3.481430293	-1.293000238	4.44E-06	0.004896974
ENSG00000148677.6	ANKRD1	-2.307361013	10.69011064	4.57E-06	0.004944149
ENSG00000145321.13	GC	1.666567337	7.349195218	5.33E-06	0.005669561
ENSG00000146469.13	VIP	-4.123260006	-1.904575575	5.62E-06	0.00587003
ENSG00000276019.1	RF02101	3.273884587	-1.553400338	6.28E-06	0.006447841
ENSG00000168334.9	XIRP1	-1.936489214	9.080349545	7.16E-06	0.007231322
ENSG00000261371.6	PECAM1	2.201058318	3.889242968	7.36E-06	0.007313689
ENSG00000172482.5	AGXT	1.956034332	4.30375696	9.10E-06	0.008895222
ENSG00000121454.6	LHX4	3.037909117	3.418703532	9.28E-06	0.008923646
ENSG00000253230.9	LINC00599	4.011196749	2.308530226	9.69E-06	0.009172017
ENSG00000110245.12	APOC3	1.552703938	6.233787548	1.00E-05	0.009361519
ENSG00000165566.12	AMER2	1.983867886	4.614730613	1.02E-05	0.009388663
ENSG00000171766.16	GATM	1.536429455	6.263267501	1.23E-05	0.01112017
ENSG00000099937.11	SERPIND1	1.542522955	5.886667873	1.36E-05	0.012113094
ENSG00000134438.10	RAX	1.762908119	4.865377726	1.51E-05	0.013279772
ENSG00000171388.12	APLN	1.66478558	4.999860787	1.57E-05	0.01362627
ENSG00000265203.2	RBP3	3.423408633	4.063698107	1.83E-05	0.015612918
ENSG00000158874.11	APOA2	1.552092783	7.367801948	2.16E-05	0.017866841
ENSG00000159409.15	CELF3	2.297201774	3.687008599	2.17E-05	0.017866841
ENSG00000188004.10	SNHG28	-6.329667526	-1.940404254	2.20E-05	0.017866841
ENSG00000173334.4	TRIB1	-1.504286367	6.813251179	2.21E-05	0.017866841
ENSG00000156427.8	FGF18	-1.514418299	5.634720695	2.51E-05	0.019984385
ENSG00000116690.12	PRG4	1.786487475	4.47767591	2.55E-05	0.020085907
ENSG00000188488.14	SERPINA5	1.470945183	6.170445662	2.79E-05	0.021649695
ENSG00000148702.15	HABP2	1.471917638	6.068026372	2.86E-05	0.021927393
ENSG00000198842.10	DUSP27	-1.563475201	7.71271023	3.13E-05	0.023683851
ENSG00000269900.3	RMRP	6.164354464	-1.439138404	3.24E-05	0.0242468
ENSG00000259753.1	AC068234.1	-7.110398881	-2.502007524	3.54E-05	0.026169877
ENSG00000243414.5	TICAM2	3.073314117	-1.45008758	3.68E-05	0.026878834
ENSG00000129521.14	EGLN3	-1.447228246	6.587331243	3.95E-05	0.028529929
ENSG00000259316.11	AC087632.1	-3.968884012	-0.377127862	4.20E-05	0.029658896
ENSG00000137673.9	MMP7	-2.309761725	3.399006277	4.21E-05	0.029658896
ENSG00000184144.12	CNTN2	2.412999753	3.286238668	4.28E-05	0.029843979
ENSG00000280571.2	AC099329.3	6.266003124	-0.860476987	4.42E-05	0.030433065
ENSG00000120738.8	EGR1	-1.478448244	7.276310938	4.54E-05	0.030908288
ENSG00000116703.14	PDC	4.349556262	3.277254978	4.85E-05	0.032658994
ENSG00000256321.6	AC087258.1	-6.007046385	-1.530546735	5.24E-05	0.034664963
ENSG00000237361.3	TUSC8	2.835375704	-1.126748187	5.26E-05	0.034664963
ENSG00000263020.6	AL662899.2	4.27346285	0.035113778	5.39E-05	0.035131349
ENSG00000015592.16	STMN4	2.619718372	3.083582247	5.74E-05	0.036989497
ENSG00000062282.15	DGAT2	1.633781438	4.642137248	6.30E-05	0.040165597
ENSG00000137193.14	PIM1	-1.515501882	5.106394517	6.52E-05	0.040506896
ENSG00000226453.2	LINC02542	3.203672669	-1.305159457	6.54E-05	0.040506896
ENSG00000151790.9	TDO2	1.768115252	4.253615555	6.55E-05	0.040506896
ENSG00000176435.7	CLEC14A	3.029046348	-0.963366482	6.67E-05	0.040816983
ENSG00000188338.15	SLC38A3	1.558602447	4.824105495	7.81E-05	0.047321607
ENSG00000184113.9	CLDN5	2.495243744	3.042330516	8.26E-05	0.049562991

* Fold change; ** counts per million; ***false discovery rate.

Table S4. DEGs detected in TSP8-15 ECTCs in response to application of rapamycin.

Ensembl ID	NCBI Gene Symbol	Log (FC) *	Log (CPM) **	P-value	FDR***
ENSG00000248713.1	C4orf54	-4.664898451	3.400444177	2.30E-07	0.012017501
ENSG00000286030.2	AC015911.9	-6.220013603	-2.236505782	4.61E-07	0.012017501
ENSG00000254692.1	AC097637.1	6.666747014	-2.646527094	8.76E-07	0.012017501
ENSG00000136881.11	AL136295.1	4.618456822	3.978110343	8.90E-07	0.012017501
ENSG00000134160.13	BAAT	4.221756709	-1.421356871	9.91E-07	0.012017501
ENSG00000115474.7	KCNJ13	5.463221818	-1.154041184	1.32E-06	0.012957713
ENSG00000173366.11	AC097637.1	-5.928520855	-2.549456817	1.83E-06	0.012957713
ENSG00000280571.2	AC006059.2	8.746782708	-0.860476987	1.92E-06	0.012957713
ENSG00000235927.4	NEXN-AS1	-3.67944954	1.770884842	2.09E-06	0.012957713
ENSG00000165828.15	PRAP1	6.449474909	1.359806072	2.14E-06	0.012957713
ENSG00000171819.5	ANGPTL7	3.462826168	-0.901459075	2.56E-06	0.014121379
ENSG00000092607.15	TBX15	2.792216321	1.936211505	2.98E-06	0.015069672
ENSG00000183160.9	TMEM119	2.512816007	3.611207451	6.02E-06	0.028083661
ENSG00000187908.19	DMBT1	2.478477184	-1.153027232	8.88E-06	0.03640958
ENSG00000151655.19	ITIH2	4.195140852	7.066942475	9.16E-06	0.03640958
ENSG00000178397.13	FAM220A	4.693854971	1.408477151	1.05E-05	0.03640958
ENSG00000130173.13	ANGPTL8	3.993307466	2.771527442	1.15E-05	0.03640958
ENSG00000080910.13	CFHR2	5.010707237	1.763767114	1.17E-05	0.03640958
ENSG00000146592.17	CREB5	-2.172000494	5.126674918	1.17E-05	0.03640958
ENSG00000287373.1	AL122014.1	2.859962876	-0.998581266	1.20E-05	0.03640958
ENSG00000251372.6	LINC00499	6.644117701	-2.405326572	1.39E-05	0.03994333
ENSG00000172482.5	AGXT	4.443982253	4.30375696	1.45E-05	0.03994333
ENSG00000254017.1	IGHEP2	-4.163078674	0.637164996	1.57E-05	0.041478766
ENSG00000197893.13	NRAP	-2.730332358	3.734621046	1.79E-05	0.042629221
ENSG00000121898.13	CPXM2	2.345974557	3.502882912	1.92E-05	0.042629221
ENSG00000223561.7	LOC646588	4.360994029	-2.107899164	1.96E-05	0.042629221
ENSG00000284779.2	AC132217.2	6.599927726	-1.001626869	1.96E-05	0.042629221
ENSG00000137869.15	CYP19A1	5.531561662	-0.159434109	1.97E-05	0.042629221
ENSG00000233639.6	PANTR1	4.111481956	-1.847882896	2.06E-05	0.043142647
ENSG00000135625.8	EGR4	-2.610910511	0.095984035	2.15E-05	0.043436346
ENSG00000072080.11	SPP2	4.608138892	0.838367362	2.47E-05	0.04828169

* Fold change; ** counts per million; ***false discovery rate.

Table S5. Heart-specific DEGs detected by comparing CC3 and TSP8-15 ECTC gene expression data sets.

Ensembl ID	NCBI Gene Symbol	Log (FC) *	Log (CPM) **	P-value	FDR***
ENSG00000109063.15	MYH3	4.806562416	5.0663884	3.16E-38	3.68E-35
ENSG00000163581.14	SLC2A2	6.05429	3.843305	4.55E-33	4.17E-30
ENSG00000133020.4	MYH8	5.05819	4.181694	7.13E-30	5.69E-27
ENSG00000137204.14	SLC22A7	4.64723	4.42816	1.21E-29	9.41E-27
ENSG00000264424.1	MYH4	6.51801	3.253815	1.50E-26	1.03E-23
ENSG00000149742.10	SLC22A9	5.952	3.34237	8.07E-25	5.09E-22
ENSG00000016490.15	CLCA1	9.23361	2.033375	1.23E-18	5.52E-16
ENSG00000171303.7	KCNK3	-3.607011	4.688356	3.65E-17	1.49E-14
ENSG00000170681.7	CAVIN4	2.56563	5.050392	1.07E-13	3.15E-11
ENSG00000265107.3	GJA5	-2.569045	5.435728	1.69E-12	4.26E-10
ENSG00000175206.10	NPPA	-3.289084	9.862624	3.20E-12	7.73E-10
ENSG00000129991.13	TNNI3	-2.429134	7.462873	2.12E-11	4.68E-09
ENSG00000196600.12	SLC22A25	7.22814	0.674748	5.03E-11	1.02E-08
ENSG00000105409.19	ATP1A3	-2.338204	5.483546	6.60E-11	1.30E-08
ENSG00000168263.9	KCNV2	-4.577793	2.936004	1.80E-10	3.27E-08
ENSG00000120937.9	NPPB	-2.043107	6.916631	3.92E-09	5.68E-07
ENSG00000130598.16	TNNI2	4.42076	2.052515	1.87E-08	2.38E-06
ENSG00000168418.7	KCNG4	-5.5184	-1.07064	3.75E-08	4.51E-06
ENSG00000113396.13	SLC27A6	-1.794155	6.594095	9.97E-08	1.11E-05
ENSG00000036448.10	MYOM2	-2.145947	4.487791	1.53E-07	1.65E-05
ENSG00000101470.10	TNNC2	3.64859	2.355678	2.14E-07	2.25E-05
ENSG00000145217.14	SLC26A1	4.28825	1.697832	3.06E-07	3.12E-05
ENSG00000156222.12	SLC28A1	4.1796	-0.24866	4.81E-07	4.72E-05
ENSG00000115474.7	KCNJ13	-3.788712	-1.15404	1.13E-06	0.000105
ENSG00000197616.12	MYH6	-2.349236	11.38822	3.90E-06	0.000326
ENSG00000138622.4	HCN4	-1.548945	7.086554	4.88E-06	0.000397
ENSG00000157087.20	ATP2B2	3.04227	2.419552	6.15E-06	0.000489
ENSG00000123104.12	ITPR2	1.77732	4.141339	8.99E-06	0.000691
ENSG00000130037.5	KCNA5	-2.559208	3.193171	1.19E-05	0.000902
ENSG00000130595.19	TNNT3	2.18894	3.32946	1.29E-05	0.000966
ENSG00000182902.14	SLC25A18	2.68316	-1.07571	1.76E-05	0.001278
ENSG00000081248.11	CACNA1S	3.89168	0.705111	2.08E-05	0.001481
ENSG00000153822.13	KCNJ16	3.81215	0.871785	2.74E-05	0.00192
ENSG00000184156.17	KCNQ3	-1.865321	3.930986	4.68E-05	0.003066
ENSG00000175003.15	SLC22A1	3.62228	0.507024	5.45E-05	0.003526
ENSG00000124134.9	KCNS1	-3.100738	2.320888	7.18E-05	0.004528
ENSG00000075429.8	CACNG5	-3.971379	0.079503	0.000121	0.007227
ENSG00000173826.14	KCNH6	-3.191873	2.124167	0.000122	0.007269
ENSG00000196296.13	ATP2A1	3.09323	1.672382	0.000127	0.007544
ENSG00000173262.11	SLC2A14	3.40909	0.653359	0.00014	0.008226
ENSG00000104848.1	KCNA7	-3.225712	-1.70215	0.000175	0.009969
ENSG00000164638.10	SLC29A4	1.61762	3.824434	0.000179	0.010161
ENSG00000078814.15	MYH7B	-1.217568	6.594421	0.000204	0.011402
ENSG00000196218.12	RYR1	1.56184	3.853993	0.000273	0.014769
ENSG00000105605.7	CACNG7	1.61853	3.713648	0.000276	0.014895
ENSG00000118729.11	CASQ2	-1.17337	6.564694	0.000334	0.0176
ENSG00000106631.8	MYL7	-1.719455	11.11759	0.000369	0.019246
ENSG00000143554.14	SLC27A3	1.3825	4.255895	0.000374	0.019427

ENSG00000007314.12	SCN4A	2.29586	-1.80231	0.00045	0.022722
ENSG00000151729.11	SLC25A4	-1.255536	8.069957	0.000494	0.024549
ENSG00000184261.4	KCNK12	-3.284161	1.068976	0.000956	0.042901
ENSG00000169432.16	SCN9A	2.02309	2.652678	0.000993	0.04407
ENSG00000117394.23	SLC2A1	-1.128446	7.670389	0.001117	0.048898

* Fold change; ** counts per million; ***false discovery rate.

Table S6. Gene ontology categories significantly altered in up-regulated group of genes in response to application of rapamycin in CC3 ECTCs.

GO Terms	Count*	GO ID	P-value**	FDR***
Biological Process				
Triglyceride metabolic process	4	6641	8.08 e^{-5}	2.1 e^{-2}
Response to oleic acid	2	34201	1.47 e^{-5}	2.29 e^{-2}
Neutral lipid catabolic process	3	46461	1.79 e^{-5}	2.29 e^{-2}
Organic acid metabolic process	8	6082	2.11 e^{-5}	2.29 e^{-2}
Neutral lipid metabolic process	4	6638	2.15 e^{-5}	2.29 e^{-2}
Regulation of very low-density lipoprotein particle remodeling	2	10901	2.2 e^{-5}	2.29 e^{-2}
Plasma lipoprotein particle clearance	3	34381	3.89 e^{-5}	2.98 e^{-2}
Amacrine cell differentiation	2	35881	4.1 e^{-5}	2.98 e^{-2}
Organic acid biosynthetic process	5	16053	4.44 e^{-5}	2.98 e^{-2}
Small-molecule metabolic process	10	44281	4.87 e^{-5}	2.98 e^{-2}
Steroid import	2	35376	5.27 e^{-5}	3.05 e^{-2}
Homeostatic process	10	42592	5.84 e^{-5}	3.2 e^{-2}
Lipid homeostasis	4	55088	6.18 e^{-5}	3.22 e^{-2}
Small-molecule biosynthetic process	6	44283	6.52 e^{-5}	3.23 e^{-2}
Glycerolipid catabolic process	3	46503	8.21 e^{-5}	3.71 e^{-2}
Neural retina development	3	3407	8.96 e^{-5}	3.8 e^{-2}
Triglyceride-rich lipoprotein particle remodeling	2	34370	9.64 e^{-5}	3.8 e^{-2}
Vesicle-mediated transport	9	16192	1.12 e^{-4}	3.8 e^{-2}
Phospholipid efflux	2	33700	1.14 e^{-4}	3.8 e^{-2}
Cellular amino acid biosynthetic process	3	8652	1.14 e^{-4}	3.8 e^{-2}
Alpha amino acid metabolic process	4	1901605	1.17 e^{-4}	3.8 e^{-2}
High-density lipoprotein particle clearance	2	34384	1.53 e^{-4}	4.74 e^{-2}
Sensory perception of light stimulus	4	50953	1.56 e^{-4}	4.74 e^{-2}
Regulation of plasma lipoprotein particle levels	3	97006	1.6 e^{-4}	4.74 e^{-2}
Aromatic amino acid family catabolic process	2	9074	1.75 e^{-4}	4.91 e^{-2}
High-density lipoprotein particle remodeling	2	34384	1.75 e^{-4}	4.91 e^{-2}
Cellular Component				
Collagen-containing extracellular matrix	6	62023	1.37 e^{-5}	2.29 e^{-2}
Spherical high-density lipoprotein particle	2	34366	4.1 e^{-5}	2.98 e^{-2}
External encapsulating structure	6	30312	6.84 e^{-5}	3.24 e^{-2}
Chylomicron	2	42627	1.14 e^{-4}	3.8 e^{-2}
Molecular Function				
Lipase inhibitor activity	3	55102	1.4 e^{-6}	1.45 e^{-2}
Lipid binding	8	8289	5.79 e^{-6}	2.1 e^{-2}
Steroid binding	4	5496	7.18 e^{-6}	2.1 e^{-2}
Organic acid binding	4	43177	3.55 e^{-5}	2.98 e^{-2}
Amino acid binding	3	16597	4.84 e^{-5}	2.98 e^{-2}
Enzyme inhibitor activity	5	4857	1.15 e^{-4}	3.8 e^{-2}

* Number of genes assigned to the GO term; ** P-value and *** FDR for gene groups were calculated by performing gene set enrichment analysis (https://www.gsea-msigdb.org/).

Table S7. Gene ontology categories significantly altered in down-regulated group of genes in CC3 ECTCs.

GO Terms	Count*	GO ID	P-value**	FDR***
Biological Process				
Tissue development	14	9888	5.16 e^{-11}	5.37 e^{-7}
Positive regulation of cell population proliferation	10	8284	1.99 e^{-9}	1.03 e^{-5}
Regulation of cell population proliferation	12	42127	3.69 e^{-9}	1.28 e^{-5}
Muscle organ development	7	7517	6.41 e^{-9}	1.67 e^{-5}
Muscle tissue development	7	60537	2.46 e^{-8}	4.89 e^{-5}
Muscle structure development	8	61061	2.82 e^{-8}	4.89 e^{-5}
Positive regulation of RNA metabolic process	11	51254	4.79 e^{-8}	7.11 e^{-5}
DNA-binding transcription activator activity	7	1216	6.21 e^{-8}	8.07 e^{-5}
Positive regulation of multicellular organismal process	10	51240	1.12 e^{-7}	1.3 e^{-4}
Positive regulation of nucleobase-containing compound metabolic process	11	45935	1.31 e^{-7}	1.37 e^{-4}
Positive regulation of cellular biosynthetic process	11	31328	1.6 e^{-7}	1.51 e^{-4}
Skeletal muscle cell differentiation	4	35914	2.04 e^{-7}	1.77 e^{-4}
Regulation of cell death	10	10941	3.14 e^{-7}	2.28 e^{-4}
Muscle cell proliferation	5	33002	1.03 e^{-6}	6.73 e^{-4}
Programmed cell death	10	10941	1.49 e^{-6}	8.64 e^{-4}
Connective tissue development	5	61448	1.81 e^{-6}	9.89 e^{-4}
Endothelial cell chemotaxis	3	35767	1.94 e^{-6}	1.01 e^{-3}
Response to endogenous stimulus	9	9719	2.62 e^{-6}	1.3 e^{-3}
Skeletal system development	6	1501	2.95 e^{-6}	1.35 e^{-3}
Circulatory system development	8	72359	3.06 e^{-6}	1.35 e^{-3}
Positive regulation of transcription by RNA polymerase II	8	45944	3.11 e^{-6}	1.35 e^{-3}
External encapsulating structure organization	5	45229	4.38 e^{-6}	1.69 e^{-3}
Response to lipids	7	33993	4.71 e^{-6}	1.75 e^{-3}
Response to corticotropin-releasing hormone	2	43435	5.95 e^{-6}	2.06 e^{-3}
Cellular response to endogenous stimulus	8	71496	7.51 e^{-6}	2.44 e^{-3}
Skeletal muscle organ development	4	60538	8.71 e^{-6}	2.74 e^{-3}
Smooth muscle cell proliferation	4	48659	1.02 e^{-5}	3.05 e^{-3}
Animal organ morphogenesis	7	9887	1.3 e^{-5}	3.65 e^{-3}
Cartilage development	4	51216	1.61 e^{-5}	4.4 e^{-3}
Response to growth factor	6	70848	1.8 e^{-5}	4.8 e^{-3}
Endothelial cell proliferation	4	1935	1.85 e^{-5}	4.8 e^{-3}
Positive regulation of epithelial cell proliferation	4	50679	2.15 e^{-5}	5.46 e^{-3}
Epithelial cell proliferation	5	50673	2.55 e^{-5}	6.3 e^{-3}
Response to oxygen-containing compound	8	1901700	3.15 e^{-5}	7.45 e^{-3}
Cellular response to oxygen-containing compound	7	1901700	3.3 e^{-5}	7.57 e^{-3}
Cardiac muscle tissue development	4	48738	3.35 e^{-5}	7.57 e^{-3}
Anatomical structure formation involved in morphogenesis	7	48646	3.47 e^{-5}	7.59 e^{-3}
Regulation of type B pancreatic cell proliferation	2	61469	3.92 e^{-5}	8.15 e^{-3}
Response to hormone	6	9725	4.36 e^{-5}	8.89 e^{-2}
Regulation of system process	5	44057	8.24 e^{-5}	1.53 e^{-2}
Cellular response to lipids	5	71396	8.45 e^{-5}	1.54 e^{-2}
Chondrocyte development	3	2063	8.94 e^{-5}	1.6 e^{-2}
Positive regulation of endothelial cell proliferation	3	1938	9.19 e^{-5}	1.62 e^{-2}

Positive regulation of cell death	5	10942	1.01 e^{-4}	1.75 e^{-2}
Enzyme-linked receptor protein signaling pathway	6	7167	1.03 e^{-4}	1.75 e^{-2}
Cell chemotaxis	4	60326	1.06 e^{-4}	1.78 e^{-2}
Response to fibroblast growth factor	3	71774	1.13 e^{-4}	1.86 e^{-2}
Type B pancreatic cell proliferation	2	44342	1.24 e^{-4}	2.02 e^{-2}
Positive regulation of protein metabolic process	7	51247	1.28 e^{-4}	2.05 e^{-2}
Negative regulation of nucleobase-containing compound metabolic process	7	45934	1.32 e^{-4}	2.08 e^{-2}
Response to molecule of bacterial origin	4	2237	1.66 e^{-4}	2.57 e^{-2}
Cell migration	7	16477	1.69 e^{-4}	2.58 e^{-2}
Tissue migration	4	90130	1.92 e^{-4}	2.81 e^{-2}
Positive regulation of gene expression	6	10628	2.36 e^{-4}	3.42 e^{-2}
Chondrocyte development	2	2063	2.73 e^{-4}	3.84 e^{-2}
Negative regulation of biosynthetic process	7	9890	2.83 e^{-4}	3.93 e^{-2}
Response to arsenic-containing substance	2	46685	3.1 e^{-4}	4.24 e^{-2}
Biomineralization	3	110148	3.28 e^{-4}	4.44 e^{-2}
MAPK cascade	5	165	3.55 e^{-4}	4.65 e^{-2}
Blood vessel endothelial cell migration	3	43534	3.57 e^{-4}	4.65 e^{-2}
Cellular Component				
External encapsulating structure	7	45229	2.5 e^{-7}	2 e^{-4}
Chromatin	8	785	4.92 e^{-6}	1.76 e^{-3}
Transcription regulator complex	5	5667	3.53 e^{-6}	7.59 e^{-3}
Contractile fiber	4	43292	3.58 e^{-5}	7.59 e^{-3}
Chromosome	8	5694	7.04 e^{-5}	1.36 e^{-2}
Collagen-containing extracellular matrix	4	62023	3.38 e^{-4}	4.5 e^{-2}
Molecular Function				
Transcription factor binding	7	8134	3.29 e^{-7}	2.28 e^{-4}
Transcription regulator activity	10	140110	1.29 e^{-6}	7.87 e^{-4}
Cis regulatory region sequence-specific DNA binding	8	987	3.69 e^{-6}	1.54 e^{-3}
Sequence-specific DNA binding	9	43565	3.97 e^{-6}	1.59 e^{-3}
RNA-polymerase-II-specific DNA-binding transcription factor binding	5	61629	7.22 e^{-6}	2.42 e^{-3}
Extracellular matrix structural constituent	4	5201	1 e^{-5}	3.05 e^{-3}
DNA-binding transcription factor activity	8	3700	1.08 e^{-5}	3.13 e^{-3}
DNA-binding transcription factor binding	5	140297	3.07 e^{-5}	7.42 e^{-3}
Glucocorticoid receptor binding	2	35259	4.63 e^{-5}	9.25 e^{-3}
Signaling receptor regulator activity	5	30545	6.14 e^{-5}	1.21 e^{-2}
cAMP response element binding	2	35497	8.05 e^{-5}	1.52 e^{-2}
Transition metal ion binding	6	46914	1.79 e^{-4}	2.66 e^{-2}
Nuclear receptor binding	3	16922	1.79 e^{-4}	2.66 e^{-2}
Hormone receptor binding	2	51427	2.73 e^{-4}	3.84 e^{-2}

* Number of genes assigned to the GO term; ** P-value and *** FDR for gene groups were calculated by performing gene set enrichment analysis (https://www.gsea-msigdb.org/).
