# Supplementary figures and images for: Contractile and Genetic Characterization of Cardiac Constructs Engineered from Human Induced Pluripotent Stem Cells: Modeling of Tuberous Sclerosis Complex and the Effects of Rapamycin
Source: Bioengineering (Basel). 2024 Feb 28;11(3):234. doi: 10.3390/bioengineering11030234 (PMC10968530; doi:10.3390/bioengineering11030234)

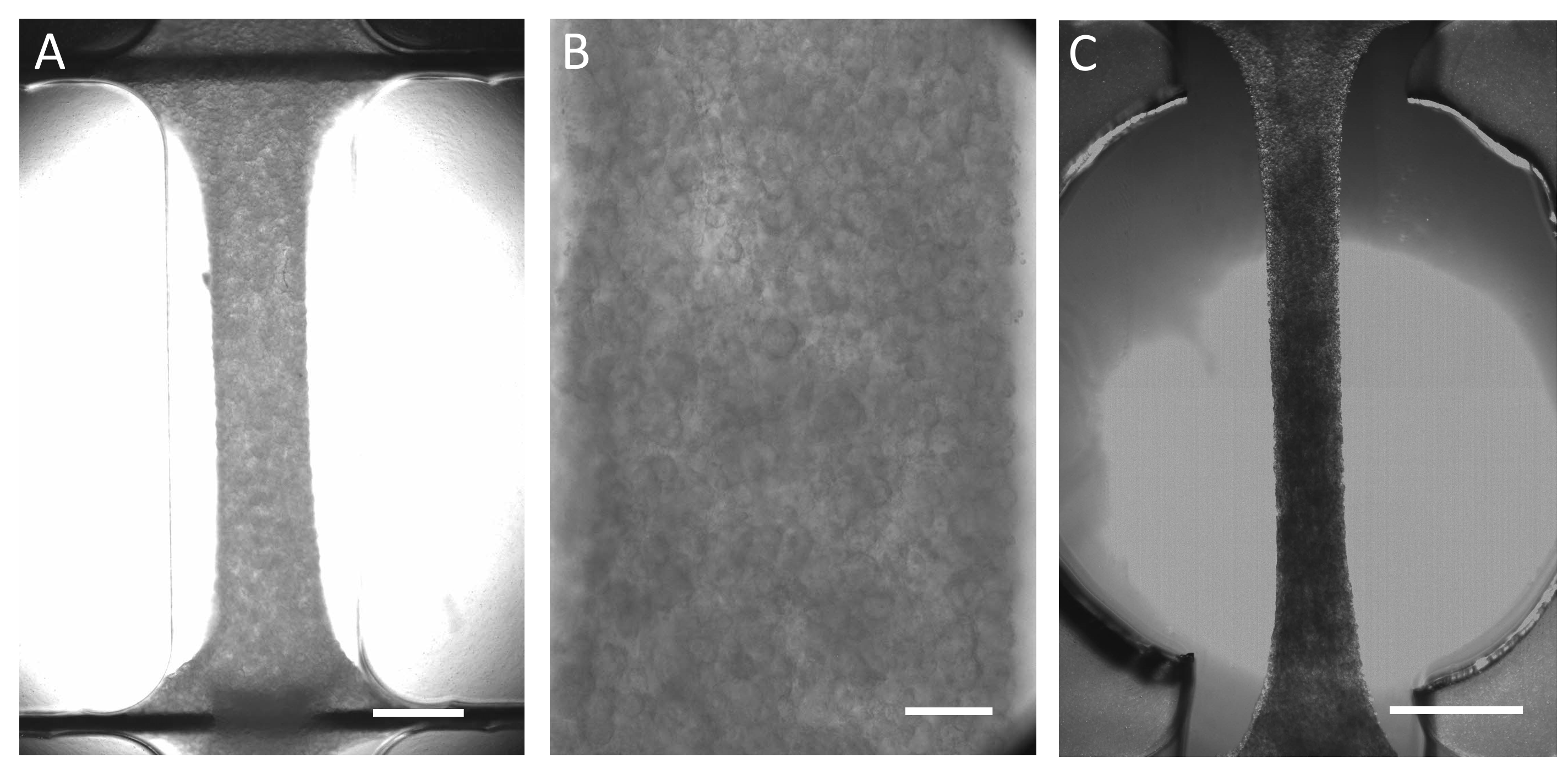

Supplement: Supplementary file 1 [file bioengineering-11-00234-s001.zip › bioengineering-2846441-supplementary/Figure S1.tif]

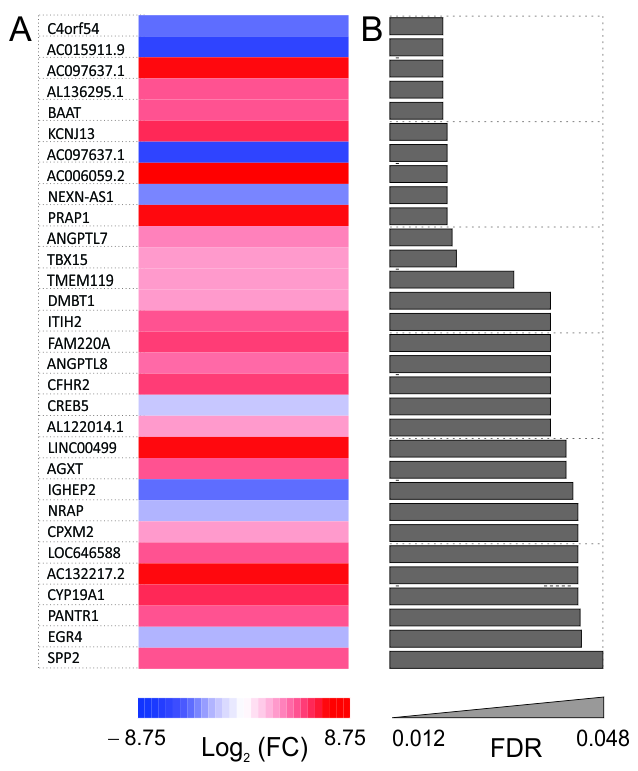

Supplement: Supplementary file 1 [file bioengineering-11-00234-s001.zip › bioengineering-2846441-supplementary/Figure S10.tif]

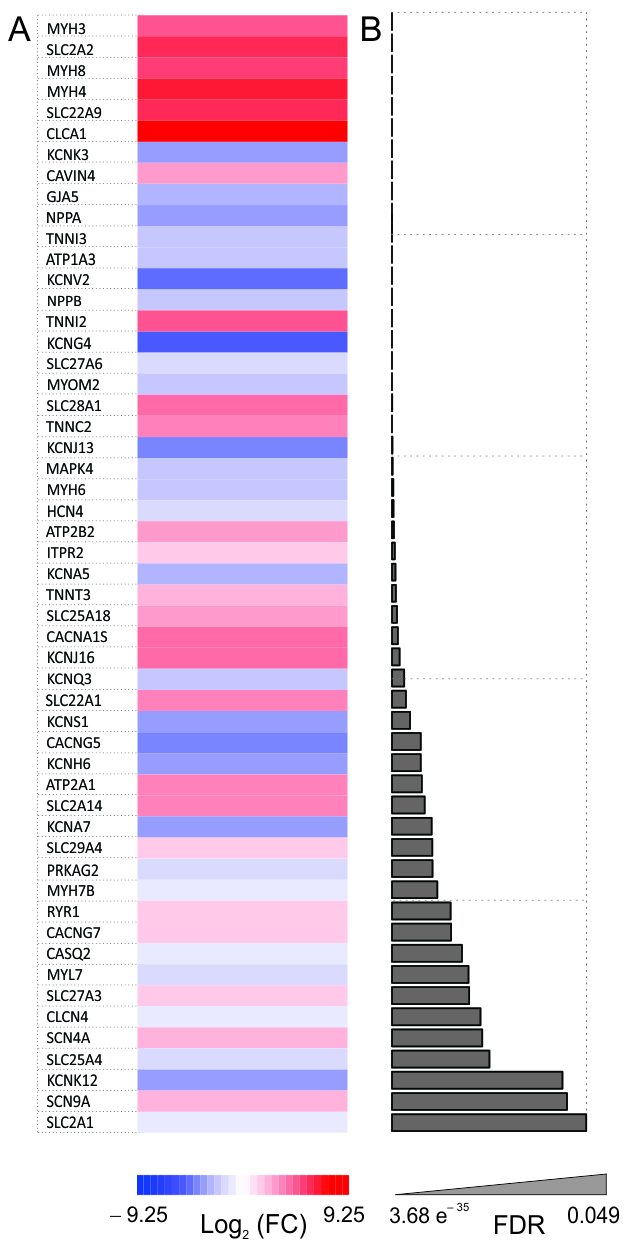

Supplement: Supplementary file 1 [file bioengineering-11-00234-s001.zip › bioengineering-2846441-supplementary/Figure S11.tif]

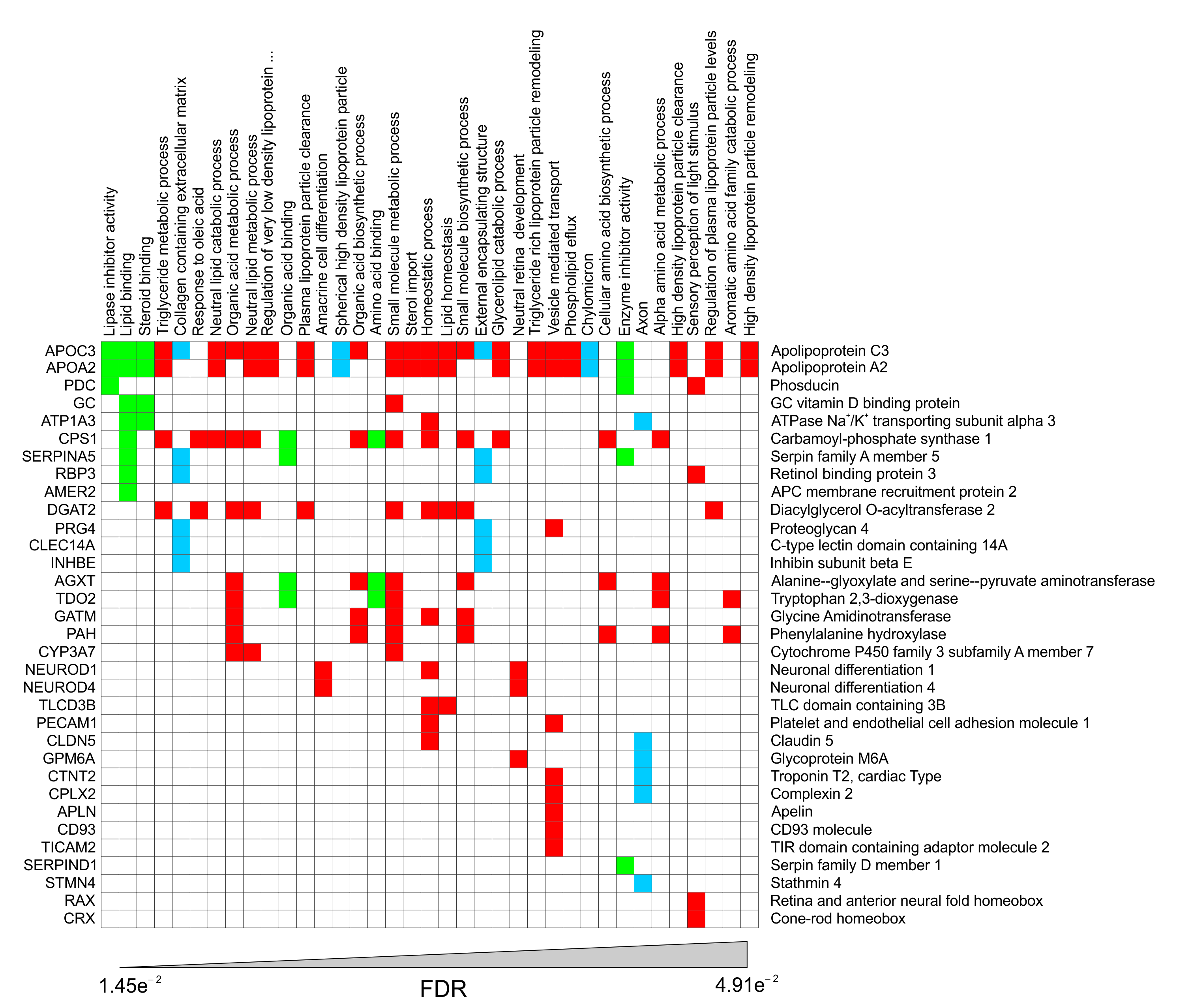

Supplement: Supplementary file 1 [file bioengineering-11-00234-s001.zip › bioengineering-2846441-supplementary/Figure S12.tif]

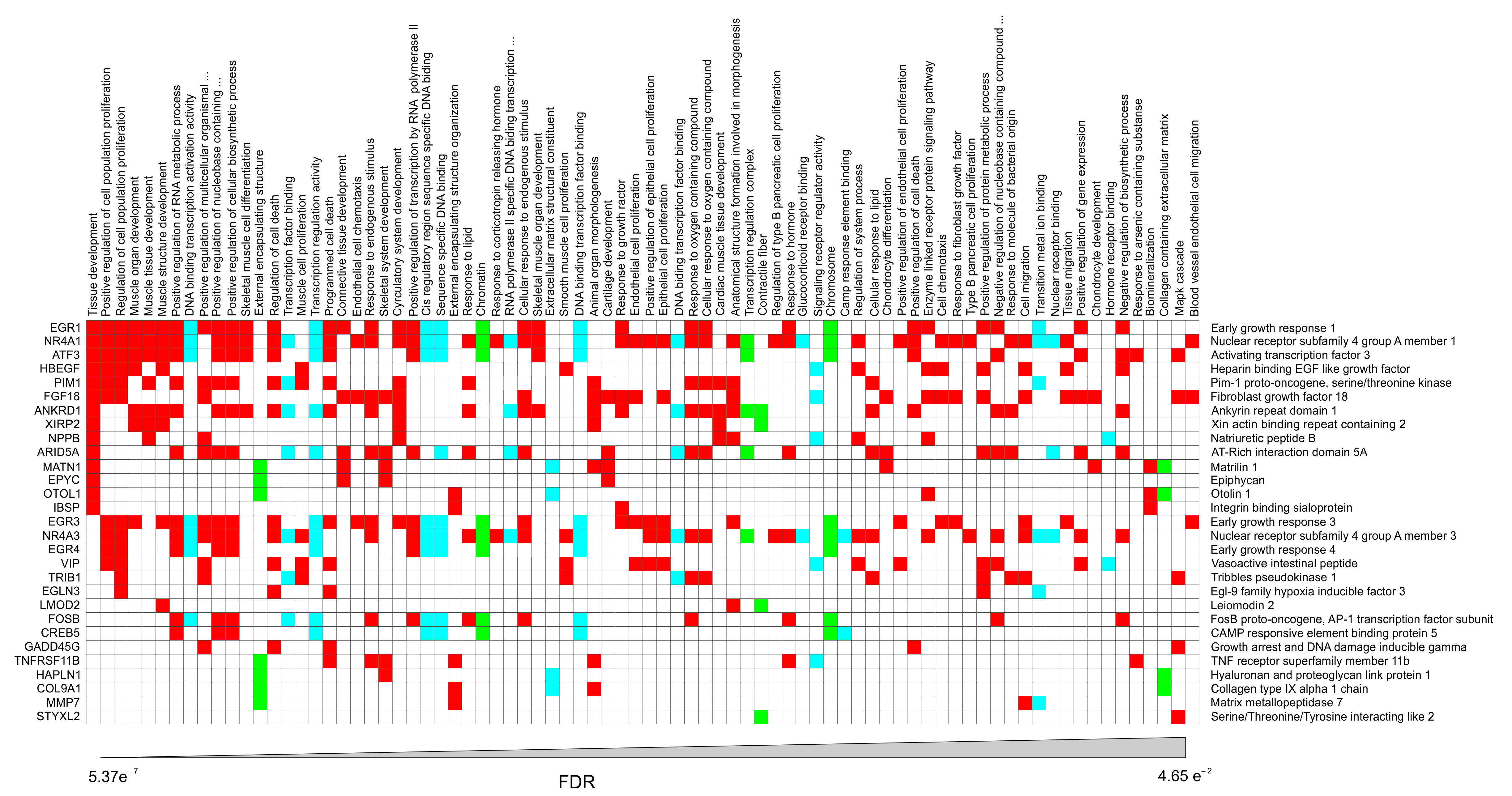

Supplement: Supplementary file 1 [file bioengineering-11-00234-s001.zip › bioengineering-2846441-supplementary/Figure S13.tif]

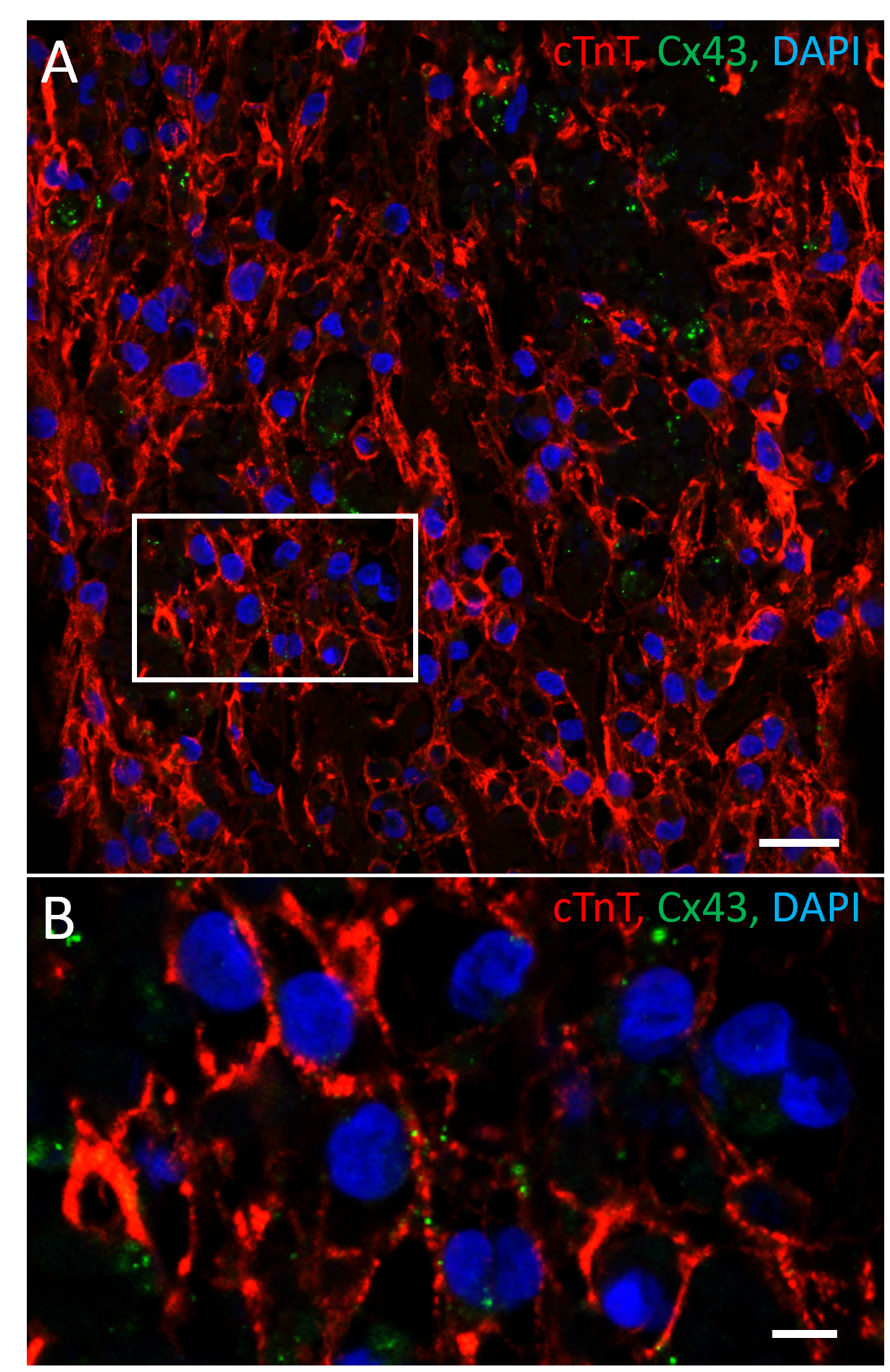

Supplement: Supplementary file 1 [file bioengineering-11-00234-s001.zip › bioengineering-2846441-supplementary/Figure S2.tif]

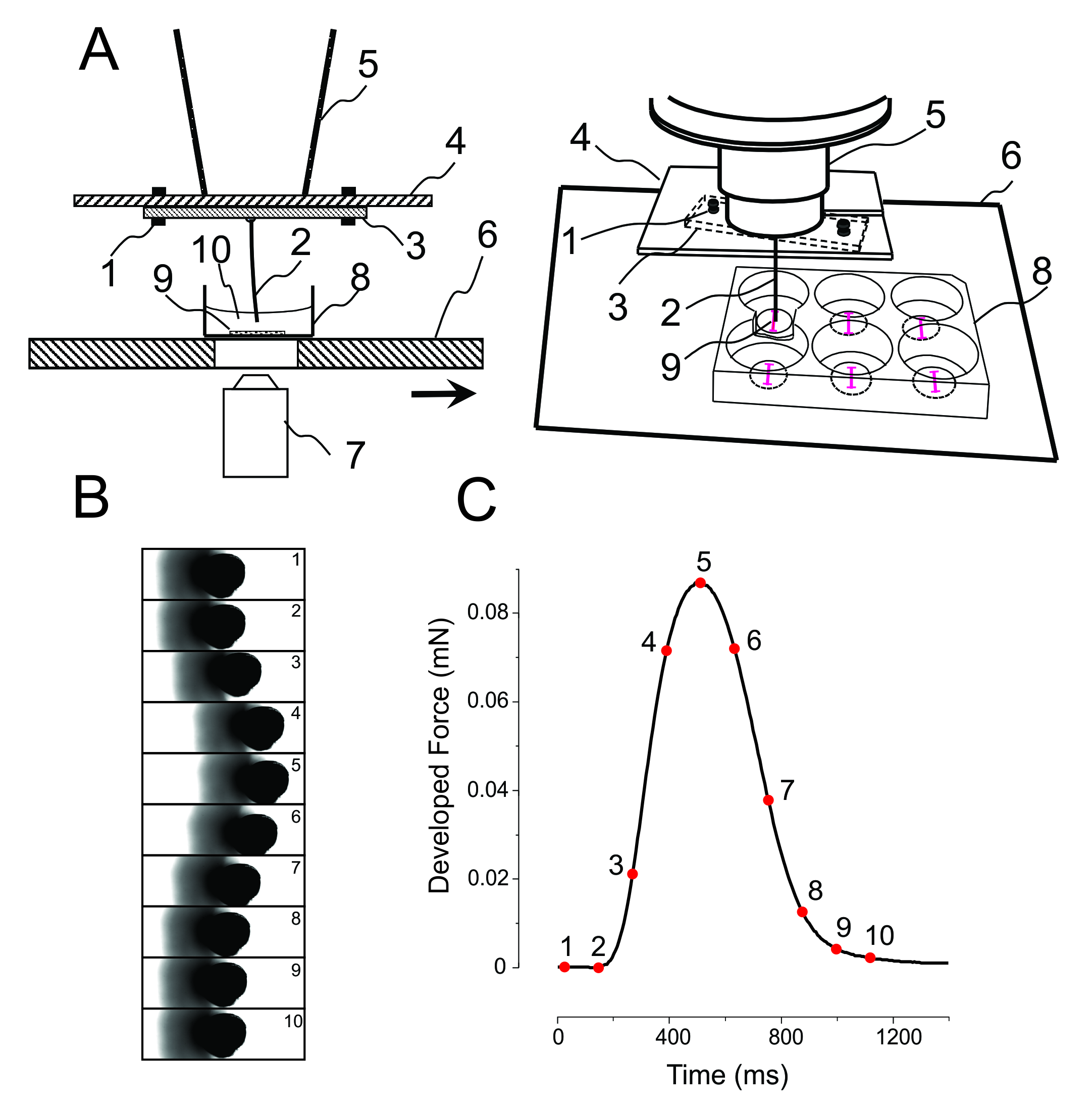

Supplement: Supplementary file 1 [file bioengineering-11-00234-s001.zip › bioengineering-2846441-supplementary/Figure S3.tif]

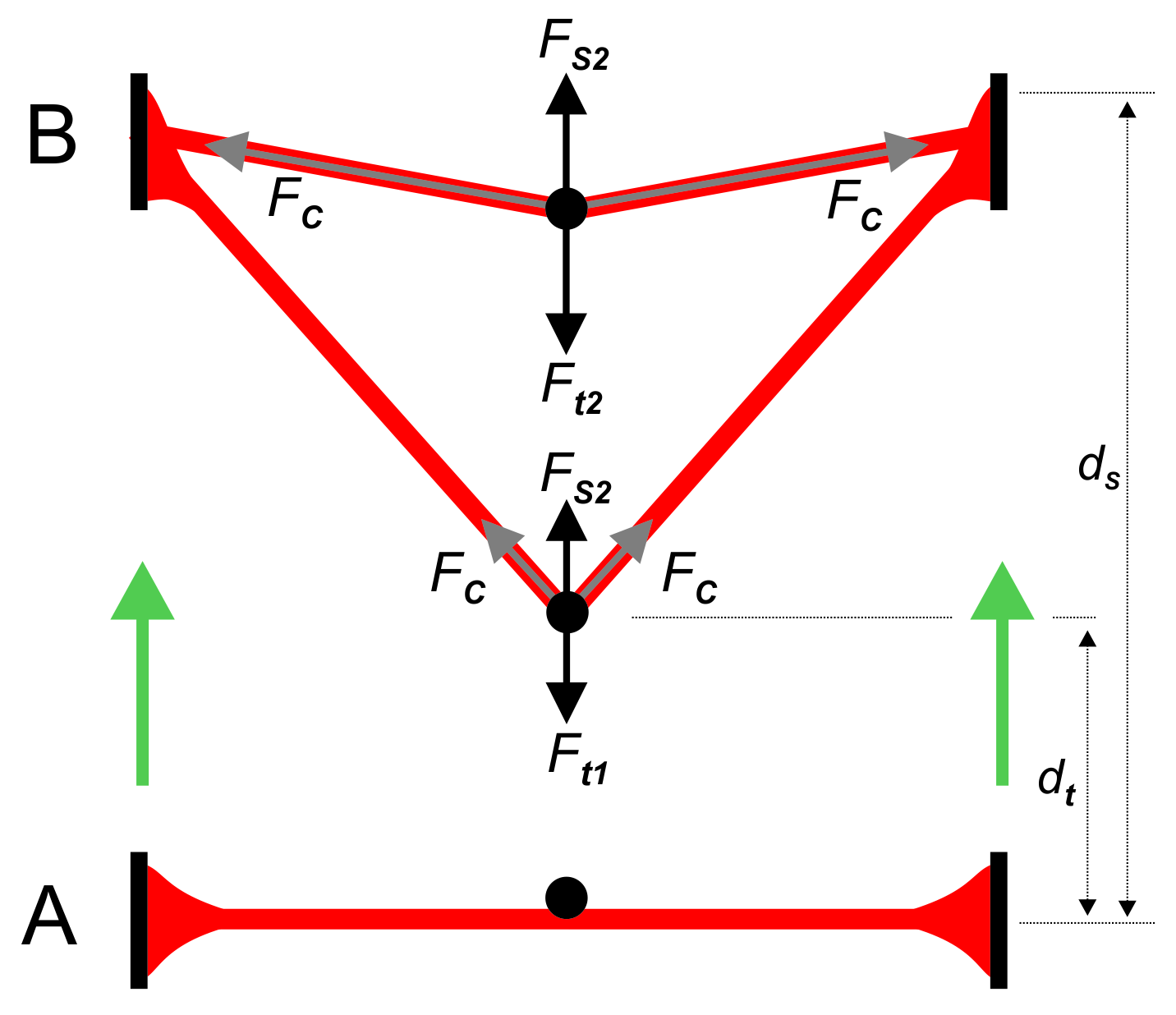

Supplement: Supplementary file 1 [file bioengineering-11-00234-s001.zip › bioengineering-2846441-supplementary/Figure S4.tif]

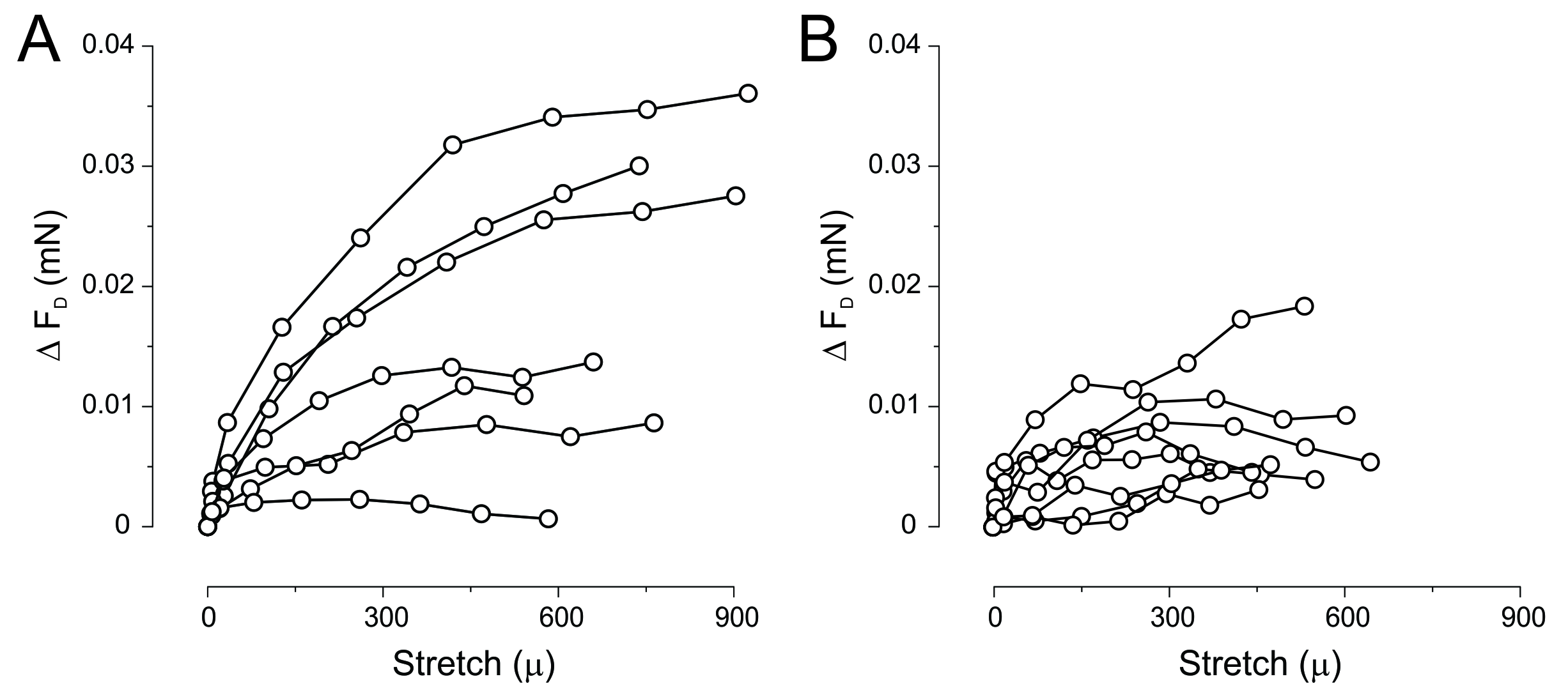

Supplement: Supplementary file 1 [file bioengineering-11-00234-s001.zip › bioengineering-2846441-supplementary/Figure S5.tif]

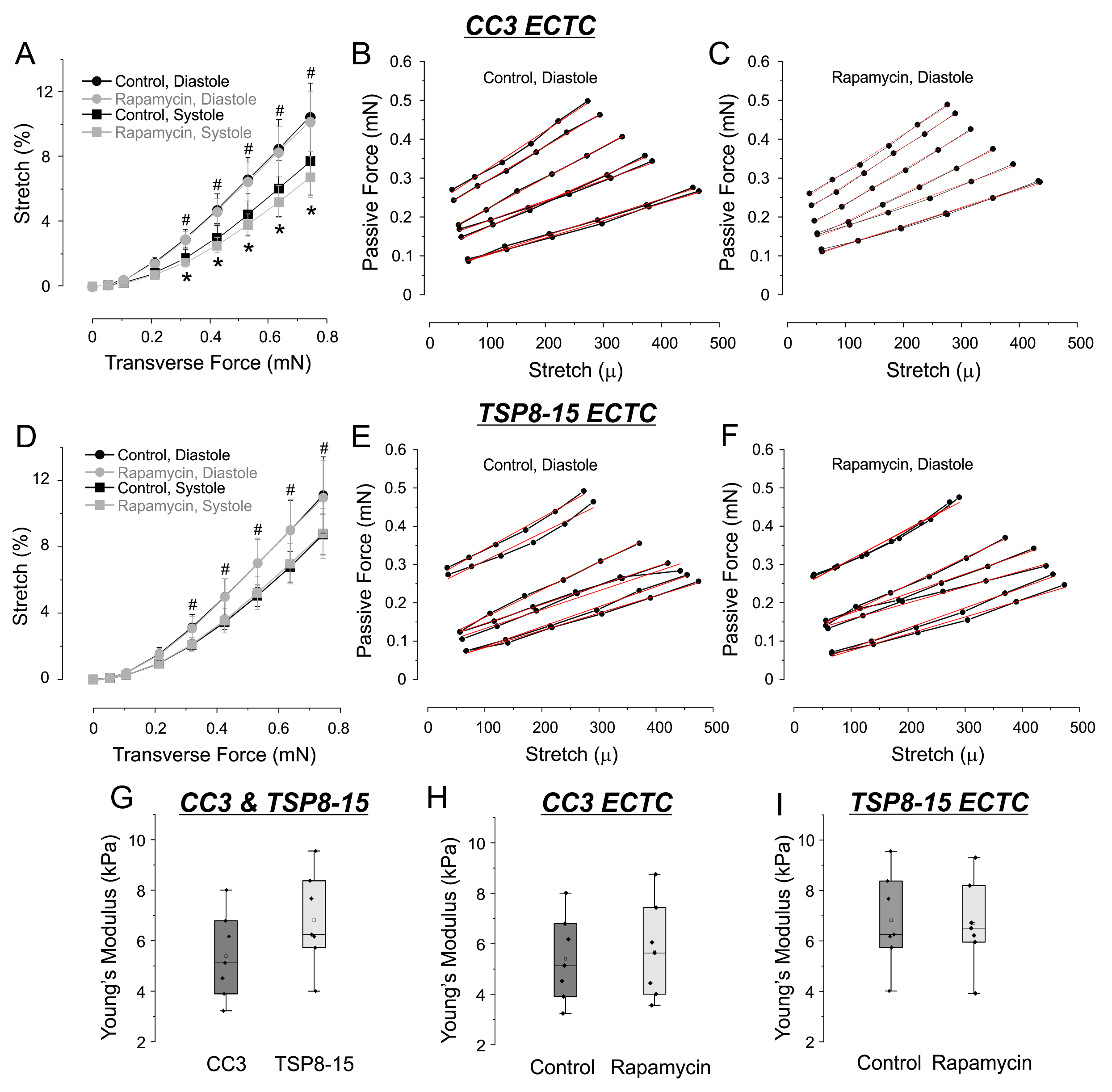

Supplement: Supplementary file 1 [file bioengineering-11-00234-s001.zip › bioengineering-2846441-supplementary/Figure S6.tif]

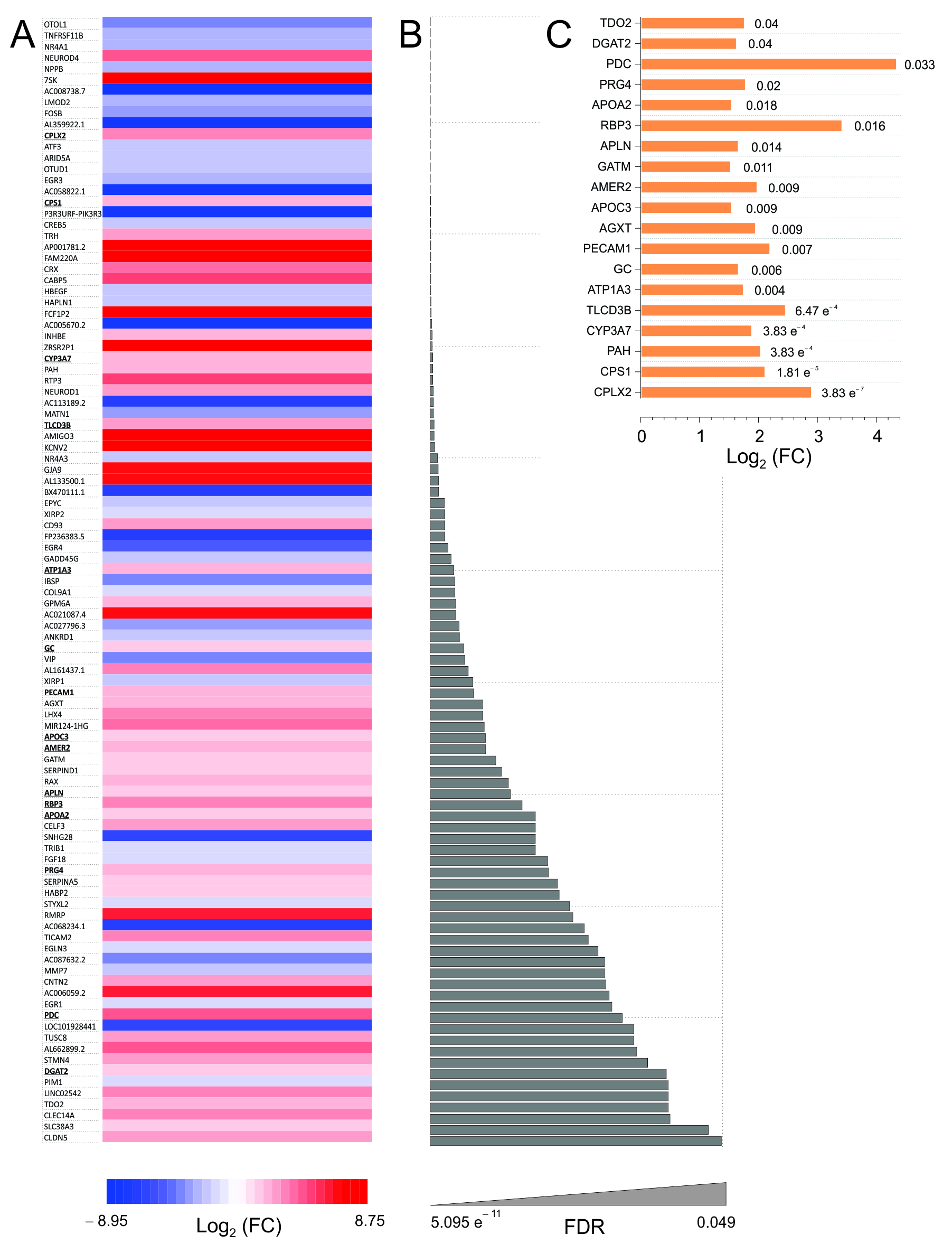

Supplement: Supplementary file 1 [file bioengineering-11-00234-s001.zip › bioengineering-2846441-supplementary/Figure S9.tif]
